# Development and temporal prospective validation of an interpretable machine learning model for identifying depressive symptoms in patients with acute exacerbation of chronic obstructive pulmonary disease

**DOI:** 10.3389/fpubh.2026.1850011

**Published:** 2026-09-08

**Authors:** Xinyue Zhou, Yu Chen, Kang Qian, Hongbin Zhu

**Affiliations:** The Fourth Affiliated Hospital of Anhui Medical University, Chaohu, China

**Keywords:** AECOPD, depression, LASSO regression, machine learning, stacked model

## Abstract

**Objective:**

To develop and temporally prospectively validate an interpretable model for identifying concurrent depressive symptoms in hospitalized patients with acute exacerbation of chronic obstructive pulmonary disease (AECOPD).

**Methods:**

This study was conducted in two stages: retrospective development and prospective validation. In the development stage, 304 AECOPD patients admitted to the Fourth Affiliated Hospital of Anhui Medical University from September 2023 to February 2025 were retrospectively enrolled and randomly divided into a training set (*n* = 214) and an internal validation set (*n* = 90) at a 7:3 ratio. Candidate predictors were screened using least absolute shrinkage and selection operator (LASSO) regression, and the selected variables were subsequently incorporated into an unpenalized multivariable logistic regression model as the primary clinical model. A nomogram was developed based on the logistic regression coefficients for individual risk estimation. Additionally, 10 machine learning algorithms were compared, and a stacked ensemble strategy was explored as a supplementary analysis to evaluate potential incremental predictive value. Model discrimination, calibration, and clinical utility were assessed using multiple metrics. In the validation stage, 120 AECOPD patients admitted to the same center from October to December 2025 were prospectively enrolled for single-center temporal validation. Model interpretability was provided by the final logistic regression coefficients and the nomogram.

**Results:**

LASSO regression screened four candidate predictors: physical function (PHYS), mental health (PSYCH), environmental support (ENVIR), and quality of life. The selected variables were entered into an unpenalized multivariable logistic regression model as the final clinical tool. The final logistic regression model achieved an *AUC* of 0.866 (95% CI: 0.819–0.912) in the training set, 0.862 in the internal validation set, and 0.866 (95% CI: 0.772–0.960) in the prospective validation set. In exploratory comparisons, the SVM + LR stacked ensemble demonstrated comparable discrimination to the logistic regression model (*AUCs*: 0.863, 0.868, and 0.864 in the training, internal validation, and prospective validation cohorts, respectively), but no statistically significant improvement was observed (DeLong test, *p* = 0.312). Calibration analysis showed good agreement in the training and internal validation sets, whereas miscalibration was observed in the prospective cohort. Decision curve analysis suggested potential clinical utility for risk stratification. Model interpretability was provided by the final logistic regression coefficients and the nomogram.

**Conclusion:**

A four-feature interpretable model was developed to identify AECOPD patients with concurrent depressive symptoms during hospitalization. The model demonstrated satisfactory discriminative performance and may assist early risk stratification; however, multicenter external validation, local recalibration, and decision-impact analysis are required before routine clinical implementation.

## 1 Introduction

Acute exacerbation of chronic obstructive pulmonary disease (AECOPD) is a critical event in the disease course, characterized by rapid worsening of respiratory symptoms that usually requires additional therapeutic interventions and is a major driver of increased hospitalization, healthcare resource consumption, and adverse clinical outcomes (1–3). Depression is one of the most common and debilitating psychological comorbidities among AECOPD patients, yet it remains largely underrecognized and undertreated (4–6). The prevalence of depressive symptoms during acute exacerbation is substantially higher than in stable COPD (4, 7, 8), and evidence suggests a bidirectional association: depression increases the risk of recurrent exacerbations and rehospitalization, while repeated exacerbations worsen depressive symptoms, creating a vicious cycle (1, 7, 9). Nationwide cohort studies have shown that AECOPD patients with comorbid depression exhibit worse functional capacity, higher respiratory symptom burden, poorer quality of life, and increased healthcare utilization (9, 10).

In clinical practice, despite widespread attention to depression screening in AECOPD patients, recognition and management of psychological comorbidities remain inadequate (11, 12). Screening tools have practical limitations, with low completion rates, resulting in many patients with comorbid depression not being timely identified and treated (2, 11). Therefore, early identification of depression in AECOPD is essential.

Based on this background, this study employed a two-stage “retrospective development + prospective validation”design, integrating multidimensional clinical data, selecting core predictive factors using LASSO, and constructing interpretable machine learning models. The aim was to provide a convenient and reliable tool for screening and risk stratification of concurrent depressive symptoms in AECOPD patients during the index hospitalization to support early identification and individualized intervention, thereby improving comprehensive management and long-term prognosis.

## 2 Materials and methods

### 2.1 Study design and participants

This study adopted a two-stage design combining retrospective model development and prospective single-center validation. A total of 304 patients with AECOPD who were treated at of the Fourth Affiliated Hospital of Anhui Medical University between September 2023 and February 2025 were retrospectively enrolled for model development. These patients were randomly divided into a training set (*n* = 214) for model construction and an internal validation set (*n* = 90) for preliminary performance evaluation at a ratio of 7:3. For prospective validation, 120 AECOPD patients treated at The Fourth Affiliated Hospital of Anhui Medical University between October and December 2025 were consecutively enrolled as an independent single-center validation cohort.

The primary outcome of this study was concurrent depressive symptom status during the index hospitalization in patients with AECOPD. The 44 candidate variables were used for initial predictor screening via LASSO regression, and the final clinical model incorporated four predictors. Sample size adequacy was evaluated for the final four-predictor unpenalized logistic regression model based on the training set (*n* = 214, 85 events, prevalence 39.7%). With a *Cox–Snell R^2^* of 0.3695 (likelihood ratio *χ^2^* = 98.71) and a desired shrinkage factor of 0.90, the minimum required sample size was calculated as:


$$\text{Nmin}=p/[R^{2}CS^{*}\;(1/S-1)]=4/[0.3695^{*}\;(1/0.90–1)]\approx 97\;\text{patients}$$

where *p* represents the number of predictor parameters in the final model. The training set of 214 patients exceeded this estimated requirement for the final logistic regression model. Given the available sample size relative to the complexity of comparing multiple machine learning algorithms and stacking combinations, these analyses were conducted as exploratory comparisons, with the parsimonious logistic regression model selected as the primary clinical tool.

Inclusion criteria included: diagnosis of COPD according to the 2023 Global Initiative for Chronic Obstructive Lung Disease (GOLD) Executive Summary, including the presence of dyspnea, chronic cough, sputum production, recurrent lower respiratory tract infections, and exposure to risk factors, with post-bronchodilator FEV1/FVC < 70% on previous or current pulmonary function testing, after exclusion of other diagnoses, and hospitalization due to acute exacerbation, defined as a short-term worsening of respiratory symptoms requiring adjustment of therapeutic management.

Exclusion criteria included: confirmed diagnosis or history of psychiatric disorders, or severe organ failure, use of antidepressants or sedative medications within the past month, impaired consciousness or language dysfunction, and visual or hearing impairment preventing completion of questionnaires, and comorbid chronic respiratory diseases other than COPD (e.g., asthma, bronchiectasis, interstitial lung disease, lung cancer, etc.).

This study was conducted in accordance with the Declaration of Helsinki and was approved by the Ethics Committee of the Fourth Affiliated Hospital of Anhui Medical University (Approval No. KYXM-202309-005). Written informed consent was obtained from all participants. For participants with cognitive impairment or incomplete civil capacity, written informed consent was obtained from their legal guardians or close relatives.

### 2.2 Data collection

Laboratory data obtained from the first examination after admission included routine blood tests (white blood cell count, neutrophil percentage and absolute count, red blood cell count, hemoglobin, platelet count), liver and renal function tests (alanine aminotransferase, aspartate aminotransferase, total bilirubin, total protein, albumin, urea, creatinine, estimated glomerular filtration rate, uric acid, blood glucose, potassium, sodium), and coagulation parameters (prothrombin time, prothrombin activity, activated partial thromboplastin time, fibrinogen, thrombin time, and D-dimer).

Baseline information was collected through structured interviews, including demographic characteristics (age, sex, educational level, smoking history (duration of smoking and number of cigarettes per day), and comorbidities), COPD-related symptom assessment using the modified Medical Research Council (mMRC) dyspnea scale, depression assessment using the Patient Health Questionnaire-9 (PHQ-9), and core scoring indices, including PHYS, PSYCH, ENVIR, social support score (SOCIL), quality of life, and health status score (Health).

Clinical information was extracted from the electronic medical record system, including the number of hospitalizations within the past year, length of the current hospital stay, use of quinolone antibiotics, use of theophylline, and corticosteroid administration (none / inhaled only / systemic use).

### 2.3 Indicator definitions and calculations

Quality of life–related dimensions: The World Health Organization Quality of Life-BREF (WHOQOL-BREF) was used to extract independent scores of the first two core dimensions, corresponding to subjective satisfaction with overall quality of life and subjective satisfaction with health status. Higher scores indicate higher satisfaction in the respective dimensions. PHYS, PSYCH, SOCIL, and ENVIR are four independent core domains of this scale, corresponding to physical function, mental health, social support, and environmental support, respectively. All items were assessed using standardized scoring, with higher scores representing better status in the corresponding dimension.

PHQ-9: Used to assess the severity of depressive symptoms over the previous 2 weeks. This scale includes nine items, each scored 0–3 based on symptom frequency, with a total score ranging from 0 to 27. Depression severity was classified as follows: 0–4, none or minimal, 5–9, mild, 10–14, moderate, 15–19, moderately severe, ≥20, severe. In this study, PHQ-9 scores were dichotomized: 0–4 as non-depression, ≥5 as depression. We used a PHQ-9 score of ≥5 to indicate at least mild depressive symptoms, which are clinically significant and have been shown to be associated with poorer health outcomes in patients with AECOPD, including increased healthcare utilization, prolonged hospitalization, reduced quality of life, and higher readmission rates (3). This threshold, encompassing patients with all severities of depressive symptoms (from mild to severe), allows our prediction model to capture the full spectrum of clinically relevant depression risk in the AECOPD population.

Social Impact Scale (SIS): Developed based on labeling theory, used to assess patients’ perceived stigma levels. Items are scored from 1 (“strongly disagree”) to 4 (“strongly agree”), with higher total scores indicating greater perceived stigma.

MMRC dyspnea scale: Used to quantify the severity of dyspnea in COPD patients, divided into five grades: 0, breathlessness only on strenuous activity, 1, shortness of breath when walking fast or climbing a small hill, 2, slower walking than peers or needing to stop due to shortness of breath, 3, stopping for breath after about 100 m or a few minutes of walking on level ground, 4, severe breathlessness preventing leaving home or causing difficulty when dressing/undressing.

### 2.4 Data preprocessing

Prior to statistical analysis and machine learning model construction, data were preprocessed. The missing rate of body mass index (BMI) exceeded 20% in both retrospective and prospective cohorts, so this variable was excluded from subsequent analysis to ensure data reliability and robustness. For other continuous variables, their missing rates were all less than 20%, and single imputation was used to handle missing values by replacing them with the overall mean of the respective variable. The same imputation parameters derived from the training set were subsequently applied to the internal validation set and prospective validation cohort without re-estimation.

### 2.5 Statistical analysis

Baseline data were described and compared using SPSS 26.0. Continuous variables with normal distribution and homogeneity of variance were presented as mean ± standard deviation and compared using independent-sample t-tests, non-normally distributed or heteroscedastic continuous variables were presented as median (*p*25–*p*75) and compared using the Mann–Whitney U test. Categorical variables were presented as [*n* (%)], with nominal variables compared using chi-square tests and ordinal variables compared using Mann–Whitney U tests.

Machine learning model construction and evaluation were performed using R software (version 4.4.1), mainly calling packages such as caret, pROC, e1071, naivebayes, xgboost, randomForest, dplyr, and glmnet. Core feature selection was performed using LASSO regression, with depression occurrence as a binary outcome. The 44 candidate variables in the training set were constructed as a matrix, *α* was set to 1, and 10-fold cross-validation was performed internally within the training set to select the regularization parameter (λ.1se = 0.0734), retaining features with non-zero regression coefficients as core predictive factors.

Based on these core features, 10 machine learning baseline models were constructed: SVM, LR, DT, PLS, GBM, NB, RF, SVC, XGB, and KNN. All baseline models were trained within the training set (*n* = 214) using repeated 5-fold cross-validation (5 folds, 3 repeats). The internal validation set and prospective validation cohort were not used during model training or parameter estimation. The classification threshold was selected from the training-set ROC analysis considering a sensitivity-oriented clinical objective to reduce missed diagnoses, and was subsequently fixed at 0.3 for evaluation in the validation cohorts. Model hyperparameters were mainly maintained according to package defaults or standard recommendations; tuning was performed only for models with explicitly specified caret tuning procedures within the training set. Key parameters for the support vector machine models were set according to package defaults or standard recommendations: linear-kernel SVM (svmLinear, C = 1.0), RBF-kernel SVC (svmRadial, C = 0.25, sigma = 0.264), and Naive Bayes (fL = 0, usekernel = FALSE, adjust = 1). LR used the default binomial logistic fitting of the glm function in the caret package, and other algorithms used either package defaults or caret-based tuning according to their model specifications. The best baseline models were selected based on *AUC*, recall, precision, and F1 score, and then used to construct stacked ensemble models. Stacked ensemble models were developed exclusively within the training set. Candidate stacking combinations were constructed using out-of-fold predicted probabilities generated by caret:trainControl(savePredictions = “final”) with 5-fold cross-validation performed exclusively within the training set. These out-of-fold predictions were used as meta-features for logistic regression meta-classifier training. The final reported stacking model combined SVM and LR as base learners. The internal validation set and prospective validation cohort were used only for model evaluation and were not involved in stacking construction.

Detailed computational specifications, including software versions, random seeds, complete tuning grids, final model objects, and stacking ensemble coefficients, are provided in Supplementary Tables S1–S3 and Supplementary File S1.

Model performance was comprehensively evaluated for discrimination, calibration, clinical utility, and interpretability. Discrimination was assessed via ROC curves and *AUC* values. Calibration was evaluated using calibration curves, the Hosmer-Lemeshow goodness-of-fit test, calibration-in-the-large (intercept), and calibration slope. No probability recalibration was performed during model development. Logistic recalibration was explored only as a post-hoc sensitivity analysis in the prospective validation cohort to evaluate potential adjustment for population differences, without modifying the original prediction model. Logistic recalibration was performed by fitting the following model:


$$\text{logit}(p_{\mathrm{cal}})=\alpha +\beta^{*}\text{logit}(p_{\mathrm{raw}})$$

where *p*_raw_ represents the original predicted probability and *p*_cal_ represents the recalibrated probability. The recalibrated results were reported only as a sensitivity analysis. Clinical utility was evaluated via DCA, and interpretability was provided by the final logistic regression coefficients and the nomogram.

All statistical analyses were two-sided, with *p* < 0.05 considered statistically significant.

## 3 Results

### 3.1 Baseline clinical and pathological characteristics of enrolled patients

**Figure 1:**
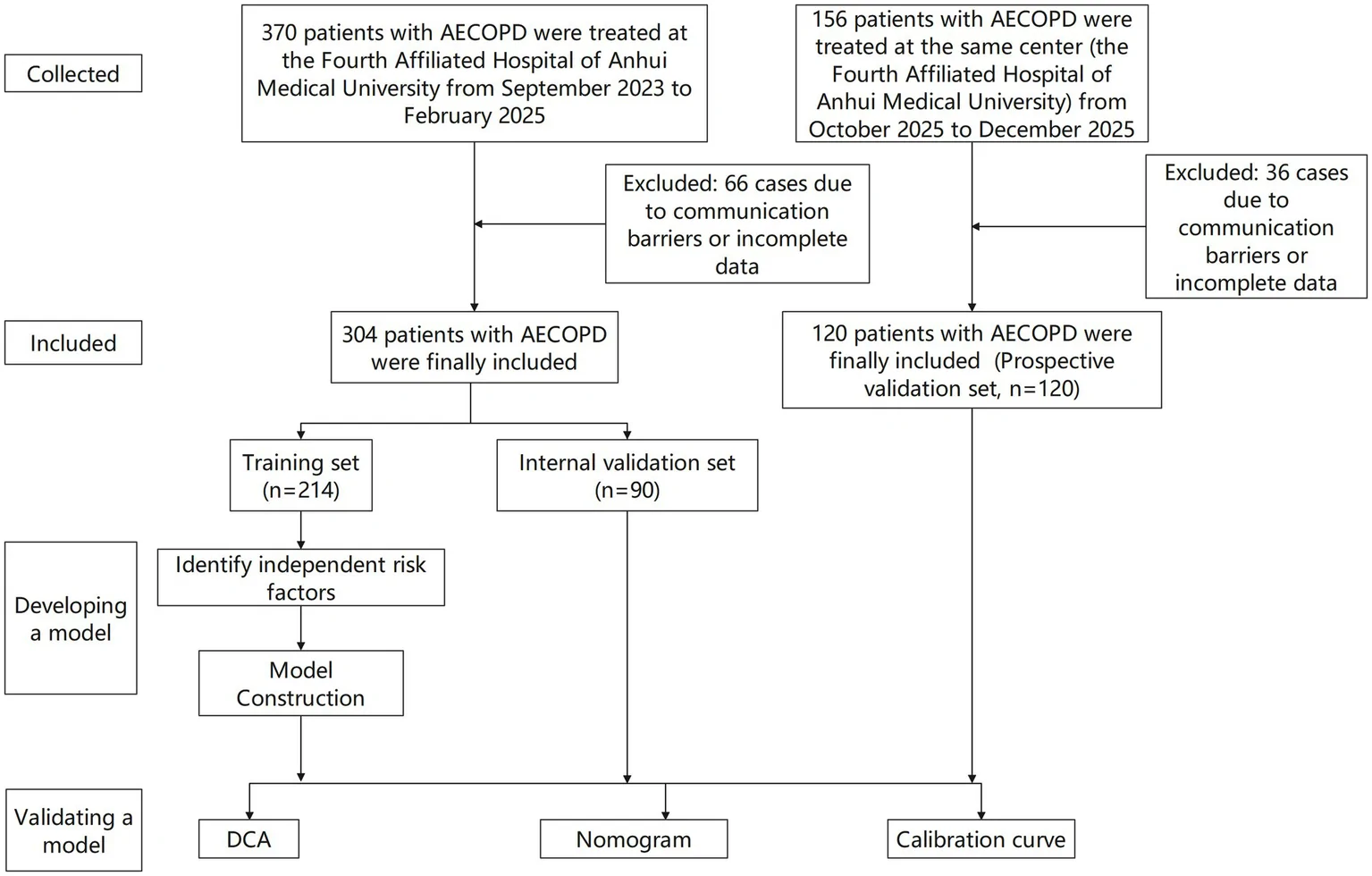
Flow chart of the study.

A total of 424 patients with AECOPD were enrolled in this study, including 304 patients in the retrospective development cohort, which were randomly divided into a training set of 214 patients and an internal validation set of 90 patients at a 7:3 ratio, and 120 patients in the prospective validation cohort, as shown in Figure 1. Depression status was determined using the PHQ-9 scale: in the retrospective cohort, 85 patients in the training set (39.7%) and 36 patients in the internal validation set (40.0%) were classified as depressed, yielding an overall depression prevalence of 39.8% (121/304), in the prospective cohort, 30 patients (25.0%) were classified as depressed.

The baseline characteristics of the retrospective development cohort and the prospective validation cohort are summarized in Table 1. The two cohorts were comparable in terms of demographic characteristics, comorbidities, and most laboratory parameters (e.g., WBC, Hb, ALT) (*p* > 0.05). Statistically significant differences were observed in smoking-related indices (smoking duration, *p* < 0.001, cigarettes per day, *p* < 0.001), certain inflammatory and coagulation parameters (Neu [%], *p* = 0.024, APTT, *p* = 0.02, FIB, *p* = 0.002, eGFR, *p* = 0.005), and core predictive features (PHYS, PSYCH, SOCIL, and ENVIR scores, all *p* < 0.001). These differences reflect the natural evolution of clinical practice at a single center over different time periods—such as intensified smoking cessation interventions, psychological support, and inflammation management in the prospective cohort—and represent genuine population heterogeneity in real-world clinical settings. This variability does not introduce selection bias and provides a realistic scenario for validating the model’s generalizability, ensuring its practical applicability in target populations subject to natural fluctuations.

**Table 1:** Comparison of baseline data between retrospective development cohort and prospective validation cohort.

Variable	Retrospective cohort (*n* = 304)	Prospective cohort (*n* = 120)	*p*-value
Sex			0.859
Male, *n* (%)	233 (77)	91 (76)	
Education level			0.074
≤ Primary school, *n* (%)	249 (82)	89 (82)	
Use of quinolones			0.698
Yes, *n* (%)	45 (15)	16 (13)	
Age, years	76.00 (71.00, 81.00)	78.00 (72.25, 83.00)	0.028
Smoking duration, years	30 (0, 50)	7.5 (0, 40)	<0.001
Cigarettes per day	20 (0, 40)	3 (0, 20)	<0.001
mMRC Grade			0.09
Grade 0, *n* (%)	0 (0)	1 (0)	
Grade 1, *n* (%)	59 (19)	3 (2)	
Grade 2, *n* (%)	66 (22)	23 (19)	
Grade 3, *n* (%)	75 (25)	62 (52)	
Grade 4, *n* (%)	104 (34)	31 (26)	
WBC (×10^9^/L)	7.60 (5.92, 10.37)	7.09 (5.62, 10.28)	0.266
Neu (%)	80.10 (70.90, 85.50)	75.90 (65.73, 84.23)	0.024
NEU (×10^9^/L)	5.97 (4.25, 8.59)	5.39 (3.72, 8.24)	0.130
RBC (×10^12^/L)	4.15 (3.73, 4.53)	4.10 (3.59, 4.52)	0.434
Hb (g/L)	126.00 (113.25, 136.75)	125.00 (113.25, 137.75)	0.718
PLT (×10^9^/L)	176.50 (139.25, 232.75)	186.50 (152.00, 230.25)	0.296
PT (s)	11.30 (10.70, 12.10)	11.30 (10.90, 11.60)	0.600
PT% (%)	89.30 (80.30, 95.90)	86.60 (82.63, 91.40)	0.101
APTT (s)	27.85 (26.00, 30.18)	27.50 (26.13, 28.58)	0.020
FIB (g/L)	4.11 (3.27, 6.49)	3.90 (3.04, 4.50)	0.002
TT (s)	17.60 (16.80, 18.60)	18.20 (17.70, 19.90)	<0.001
D-Dimer (ug/ml)	0.58 (0.35, 1.13)	0.59 (0.36, 1.09)	0.961
ALT (U/L)	16.50 (12.00, 24.75)	16.00 (12.00, 23.75)	0.333
AST (U/L)	22.50 (18.00, 30.00)	22.00 (18.00, 27.00)	0.318
TBIL (μmol/L)	12.80 (9.00, 17.00)	13.25 (9.75, 16.60)	0.576
TP (g/L)	65.05 (60.30, 70.83)	67.05 (62.30, 72.05)	0.053
ALB (g/L)	40.3 ± 5.9	40.7 ± 6.5	0.578
Urea (mmol/L)	7.00 (5.40, 9.20)	7.00 (5.51, 9.18)	0.622
Creatinine (μmol/L)	65.00 (53.93, 83.00)	71.85 (56.55, 84.98)	0.096
eGFR (ml/min/1.73m^2^)	93.30 (81.93, 100.35)	87.05 (75.80, 98.20)	0.005
Uric Acid (μmol/L)	289.50 (232.00, 384.75)	285.00 (222.50, 390.25)	0.645
GLU (mmol/L)	5.80 (5.00, 7.30)	6.19 (5.18, 7.80)	0.165
K^+^ (mmol/L)	3.874 ± 0.433	3.879 ± 0.401	0.912
Na^+^ (mmol/L)	140.50 (137.80, 142.38)	140.05 (137.73, 141.98)	0.346
Stigma (score)	52 (49, 55)	50 (41.5, 56)	0.072
PHYS (score)	11.43 (9.71, 13.14)	12.00 (11.43, 14.29)	<0.001
PSYCH (score)	12.00 (11.33, 12.67)	14.00 (12.67, 15.33)	<0.001
SOCIL (score)	12.00 (12.00, 12.00)	12.00 (12.00, 13.33)	<0.001
ENVIR (score)	13.00 (11.50, 14.50)	14.25 (13.00, 16.00)	<0.001
Quality of life			<0.001
1, *n* (%)	1 (0)	0 (0)	
2, *n* (%)	44 (14)	9 (8)	
3, *n* (%)	174 (57)	61 (51)	
4, *n* (%)	85 (28)	39 (33)	
5, *n* (%)	0 (0)	11 (9)	
Health status			0.182
1, *n* (%)	3 (1)	7 (6)	
2, *n* (%)	145 (48)	42 (35)	
3, *n* (%)	134 (44)	56 (47)	
4, *n* (%)	22 (7)	15 (13)	
5, *n* (%)	0 (0)	0 (0)	
Comorbidities			0.109
Yes, *n* (%)	212 (70)	92 (70)	
Annual hospitalizations, times	1 (1, 2)	1 (1, 2)	0.137
Hospital stay, days	8 (7, 10)	8 (6, 10)	0.127
Use of theophylline			0.324
Yes, *n* (%)	246 (81)	102 (85)	
Use of corticosteroids			0.125
No, *n* (%)	23 (8)	4 (3)	
Inhaled only, *n* (%)	48 (16)	22 (12)	
Systemic, *n* (%)	233 (76)	90 (75)	

Comorbidities refer to hypertension, diabetes mellitus, coronary atherosclerotic heart disease, and cerebral infarction. WBC, White Blood Cell Count; Neu, Neutrophil Percentage; NEU, Absolute Neutrophil Count; RBC, Red Blood Cell Count; Hb, Hemoglobin; PLT, Platelet Count; PT, Prothrombin Time; PT%, Prothrombin Time Activity; APTT, Activated Partial Thromboplastin Time; FIB, Fibrinogen; TT, Thrombin Time; D-Dimer, D-Dimer; ALT, Alanine Aminotransferase; AST, Aspartate Aminotransferase; TBIL, Total Bilirubin; TP, Total Protein; ALB, Albumin, Urea, Urea, Creatinine, Creatinine; eGFR, Estimated Glomerular Filtration Rate; Uric Acid, Uric Acid; GLU, Blood Glucose; K^+^, Potassium; Na^+^, Sodium.

Additional abbreviations for core predictive features: PHYS, physical function domain; PSYCH, mental health domain; SOCIL, social support domain; ENVIR, environmental support domain (all from WHOQOL-BREF). Domain scores were calculated by taking the mean of the domain items and multiplying by 4, resulting in a transformed score ranging from 4 to 20 (22).

*p* < 0.05 was considered statistically significant.

The comparison of baseline characteristics between the training set and internal validation set is presented in Supplementary Table S4. Statistical analyses showed that the two sets were well balanced across most variables, including demographics, comorbidities, laboratory indices, and core predictive features (*p* > 0.05), with the exception of corticosteroid administration (*p* = 0.008). These results indicate good internal homogeneity within the retrospective development cohort, supporting the rationale for the 7:3 split for model training and internal validation without introducing significant selection bias.

### 3.2 Selection of predictive factors

In this study, the depressive status of AECOPD patients in the training set was treated as a binary outcome variable, and 44 candidate clinical indicators were used as independent variables. LASSO regression (*α* = 1) was applied to select predictive factors. Ten-fold cross-validation was used to determine a more robust regularization parameter, with *λ_1se_* = 0.0734 (selected via the 1-standard error rule) was chosen as the optimal penalty parameter to balance model complexity and generalizability based on a balance between model complexity and generalizability.

**Figure 2:**
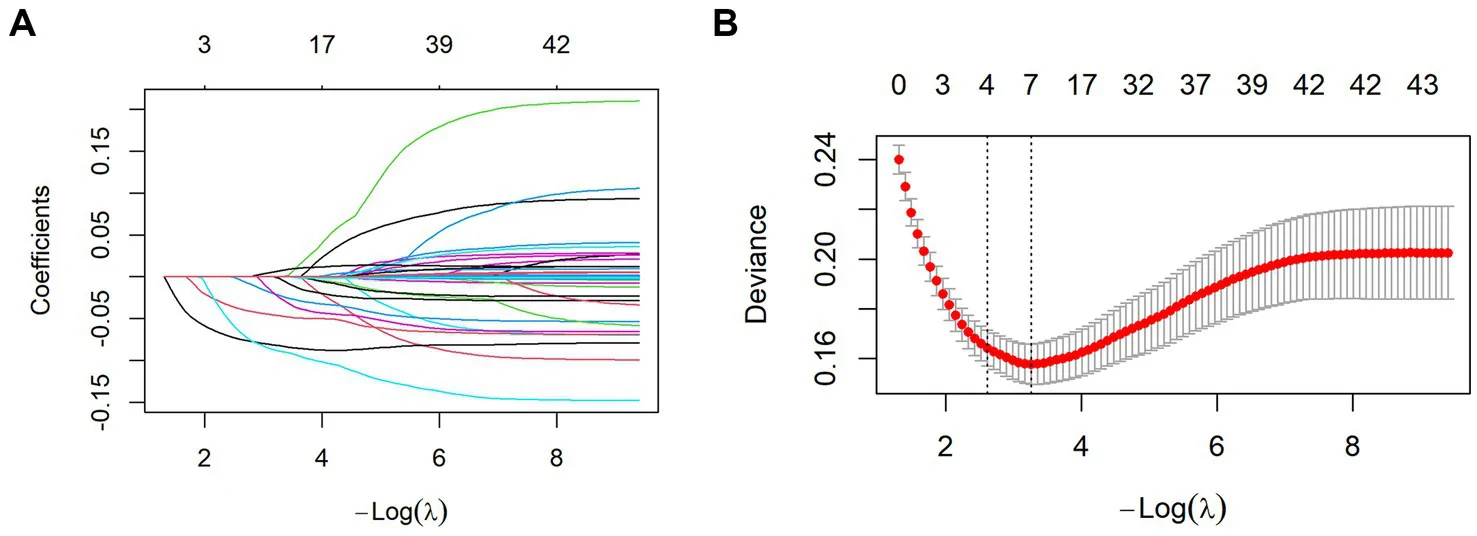
LASSO regression analysis for variable selection. LASSO regression coefficient path plot **(A)** and LASSO regression cross-validation curve **(B)**.

The selection results identified four core predictive factors with non-zero regression coefficients: PHYS, PSYCH, ENVIR, and quality of life, with corresponding coefficients of −0.074711, −0.038484, −0.003864, and −0.066359, respectively. All core predictive factors had negative coefficients, indicating that lower scores on these indicators were associated with a higher risk of depression in patients with AECOPD. The relevant coefficient paths are shown in Figure 2.

### 3.3 Construction of machine learning prediction models

Based on the four core features, 10 baseline machine learning models including DT and PLS were constructed. All models were trained using 5-fold cross-validation repeated three times. The classification decision threshold was uniformly set at 0.3, and model selection was primarily based on *AUC*, supplemented by multiple performance metrics.

#### 3.3.1 Training of individual models

The presence of depression in AECOPD patients was treated as a binary outcome variable, with the four core features as independent variables. The decision threshold was set to 0.3, and *AUC* was the primary evaluation metric. Additional performance metrics including accuracy, recall, precision, sensitivity, specificity, F1 score, area under the receiver operating characteristic curve, and Brier score were simultaneously recorded to comprehensively quantify model performance.

#### 3.3.2 Model performance evaluation

**Figure 3:**
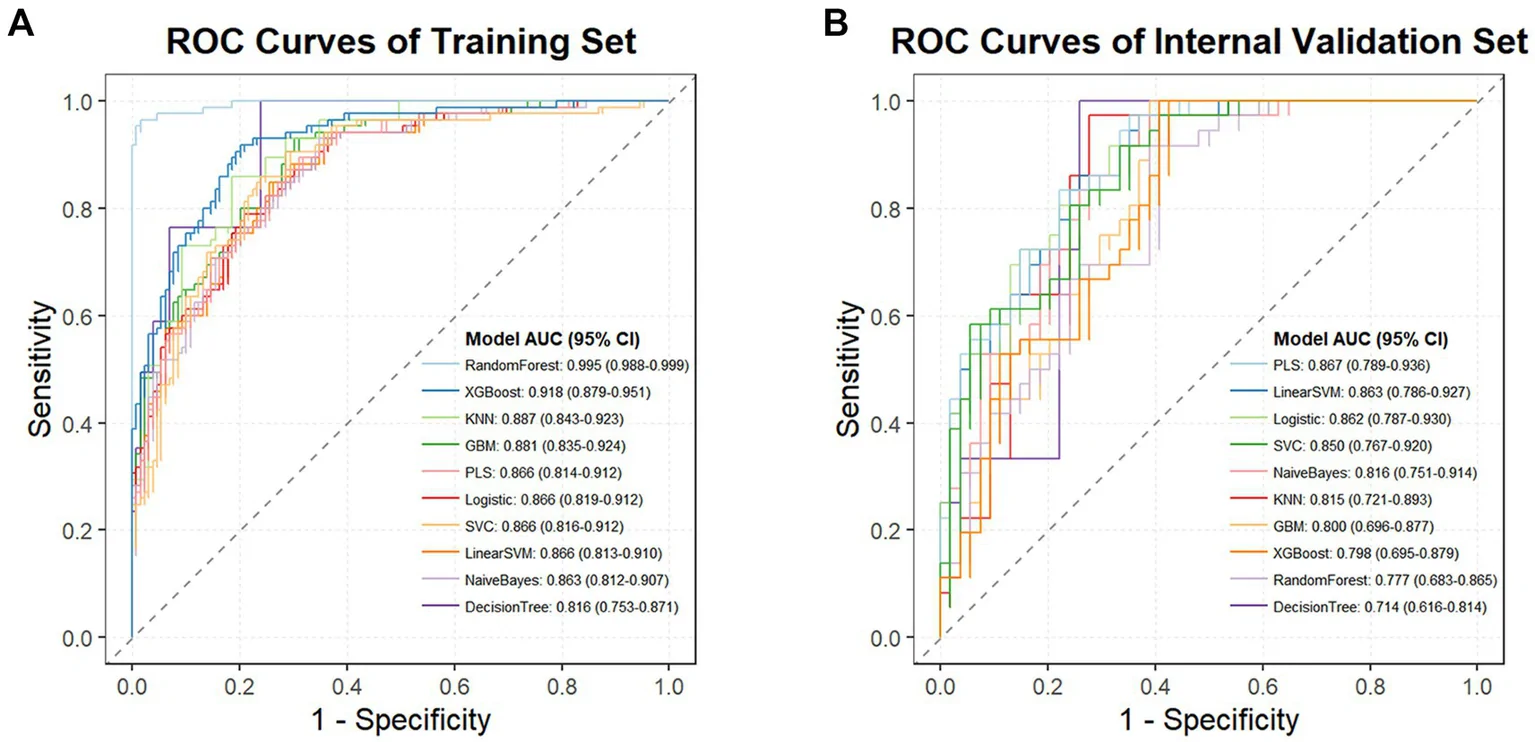
**(A)** ROC curve for the training set and **(B)** ROC curve for the internal validation set.

The core performance metrics of the 10 models on the internal validation set are shown in Table 2, while training set metrics are presented in Supplementary Table S5. Results from the internal validation set indicated that five models, SVM, LR, NB, SVC, and XGB, performed satisfactorily with *AUC*s of 0.863, 0.862, 0.815, 0.801, and 0.798, respectively, and both accuracy and F1 scores above 0.68, with no single metric showing a major deficiency. RF exhibited typical overfitting, with an *AUC* of 0.995 in the training set but dropping sharply to 0.777 in the internal validation set. Although PLS reached an *AUC* of 0.867, its accuracy was only 0.544 and specificity 0.241, indicating severely imbalanced performance (Figure 3).

**Table 2:** Core performance metrics of various machine learning models on the internal validation set.

ML	Accuracy	Recall	Precision	Specificity	F1 score	*AUC*	Brier score
DT	0.733	0.722	0.65	0.741	0.684	0.714	0.223
PLS	0.544	1	0.468	0.241	0.637	0.867	0.183
GBM	0.678	0.778	0.571	0.611	0.659	0.8	0.186
SVM	0.744	0.861	0.632	0.667	0.729	0.863	0.152
LR	0.767	0.861	0.66	0.704	0.747	0.862	0.151
NB	0.733	0.889	0.615	0.63	0.727	0.816	0.189
RF	0.644	0.694	0.543	0.611	0.61	0.777	0.201
SVC	0.767	0.806	0.674	0.741	0.734	0.85	0.166
XGB	0.711	0.806	0.604	0.648	0.69	0.798	0.185
KNN	0.711	0.917	0.589	0.574	0.717	0.815	0.171

To visually present model discrimination performance, ROC curves were plotted for the training set (Figure 3A) and the internal validation set (Figure 3B). The curves used 1 minus specificity as the x-axis and sensitivity as the y-axis, with the diagonal line representing random chance. The *AUC* values of each model and their 95% confidence intervals were annotated.

#### 3.3.3 Stacked ensemble model construction

SVM, LR, and NB were selected as base models based on performance standards and complementary characteristics. All three models performed excellently individually (*AUC* > 0.81). SVM excels at capturing nonlinear relationships, LR offers strong interpretability and stable generalization, and NB is highly efficient in probability generation. The characteristics of these algorithms complement each other effectively, with no risk of overfitting.

XGBoost is a gradient-boosted tree ensemble that partitions feature spaces through sequential decision trees, whereas SVM and SVC are margin-based kernel methods; these families are algorithmically distinct. SVM (linear kernel, svmLinear) and SVC (RBF kernel, svmRadial) differ only in kernel function. All 10 baseline models were retained as independent candidates and evaluated separately. The final reported stacking ensemble selected SVM and LR as base learners based on empirical performance and complementary characteristics, not because any algorithm was redundant.

A stacked ensemble strategy was adopted to combine the three base models (SVM, LR, and NB) within a two-layer framework: a base model layer and a meta-classifier layer. The base model layer outputs the predicted probabilities of depression status from each individual model, and the meta-classifier layer uses LR to fit the final prediction result by integrating these predicted probabilities as new features, fully leveraging the advantages of different algorithms.

To prevent information leakage, the stacking ensemble was developed exclusively within the training set. Candidate base models (SVM and LR) were trained using the training data, and out-of-fold predicted probabilities were generated using 5-fold cross-validation implemented via caret:trainControl(savePredictions = “final”). These out-of-fold predictions were used as meta-features for training the logistic regression meta-classifier. The internal validation set and prospective validation cohort were never involved in stacking construction or hyperparameter optimization.

Based on the characteristics of the base models, four stacking combinations were constructed, including three pairwise stacks (SVM + LR, SVM + NB, LR + NB) and one triple-stack combination (SVM + LR + NB). The optimal solution was selected based on a comprehensive evaluation of multidimensional indicators. The core performance metrics of the four stacking combinations in the internal validation set are shown in Table 3, with training set indicators presented in Supplementary Table S6. ROC curves of the four candidate stacking combinations in the training and internal validation sets are provided in Supplementary Figure S1.

**Table 3:** Core performance metrics of different stacking ensemble models in the internal validation set.

ML	Accuracy	Recall	Precision	Specificity	F1 score	*AUC*	Brier score
LR + SVM	0.778	0.806	0.690	0.759	0.744	0.868	0.154
NB + SVM	0.744	0.778	0.651	0.722	0.709	0.805	0.178
LR + NB	0.756	0.778	0.67	0.741	0.718	0.848	0.165
LR + NB + SVM	0.744	0.778	0.651	0.722	0.709	0.832	0.167

Column names indicate the stacking model combinations: LR+SVM, LR+NB, NB+SVM, and LR+NB+SVM.

#### 3.3.4 Selection of the optimal predictive model

Among the 10 baseline algorithms, logistic regression (LR) and support vector machine (SVM) demonstrated the strongest and most balanced discriminative performance in both the training set (*AUC* 0.866 and 0.866, respectively) and the internal validation set (*AUC* 0.862 and 0.863, respectively; Table 2). Consequently, these two algorithms were selected as candidate base learners, and an exploratory SVM + LR stacking ensemble was constructed for head-to-head comparison.

In the training set, the LR model achieved an *AUC* of 0.866 and a Brier score of 0.146, whereas the stacking ensemble achieved an *AUC* of 0.863 and a Brier score of 0.149. In the internal validation set, the LR model achieved an *AUC* of 0.862 and a Brier score of 0.151, compared with 0.868 and 0.154 for the stacking ensemble. The incremental improvement of stacking over LR was marginal (Δ*AUC* ≤ 0.006) and did not reach statistical significance in either cohort (*p* > 0.30). Given the comparable discriminative performance, the selection of the final clinical prediction tool was deferred to prospective validation; accordingly, both models were independently evaluated in the temporal prospective cohort.

#### 3.3.5 Prospective validation of the optimal model

Both the LR model and the exploratory SVM + LR stacking ensemble were prospectively validated in the independent temporal cohort (*n* = 120). As shown in Table 4, the two models again achieved nearly identical performance. The LR model attained an *AUC* of 0.866 (95% CI: 0.772–0.960), accuracy of 88.3%, recall of 80.0%, specificity of 91.1%, and Brier score of 0.098. The stacking ensemble achieved an *AUC* of 0.864 (95% CI: 0.770–0.958), accuracy of 89.2%, recall of 73.3%, specificity of 94.4%, and Brier score of 0.098. The difference in *AUC* was not statistically significant (*p* = 0.356).

**Table 4:** Core performance metrics in the prospective validation set.

ML	Accuracy	Recall	Precision	Specificity	F1 score	*AUC*	Brier score
LR	0.883	0.8	0.75	0.911	0.774	0.866	0.098
LR + SVM	0.892	0.733	0.815	0.944	0.772	0.864	0.098

These results confirm that both approaches maintain stable predictive performance in newly collected target populations, and that the added complexity of stacking does not translate into measurable prospective gain. The final selection of the clinical prediction tool was therefore based on subsequent calibration and clinical utility assessments.

### 3.4 Model validation and interpretability analysis

The final logistic regression model was comprehensively evaluated in terms of discriminative ability, calibration, clinical utility, and interpretability by plotting calibration curves and decision curve analysis (DCA) for the training set, internal validation set, and prospective validation set. Additionally, a nomogram was constructed to provide direct bedside interpretability of the model’s predictions. The exploratory SVM + LR stacked ensemble was assessed in parallel for comparison where applicable.

#### 3.4.1 Model calibration

**Figure 4:**
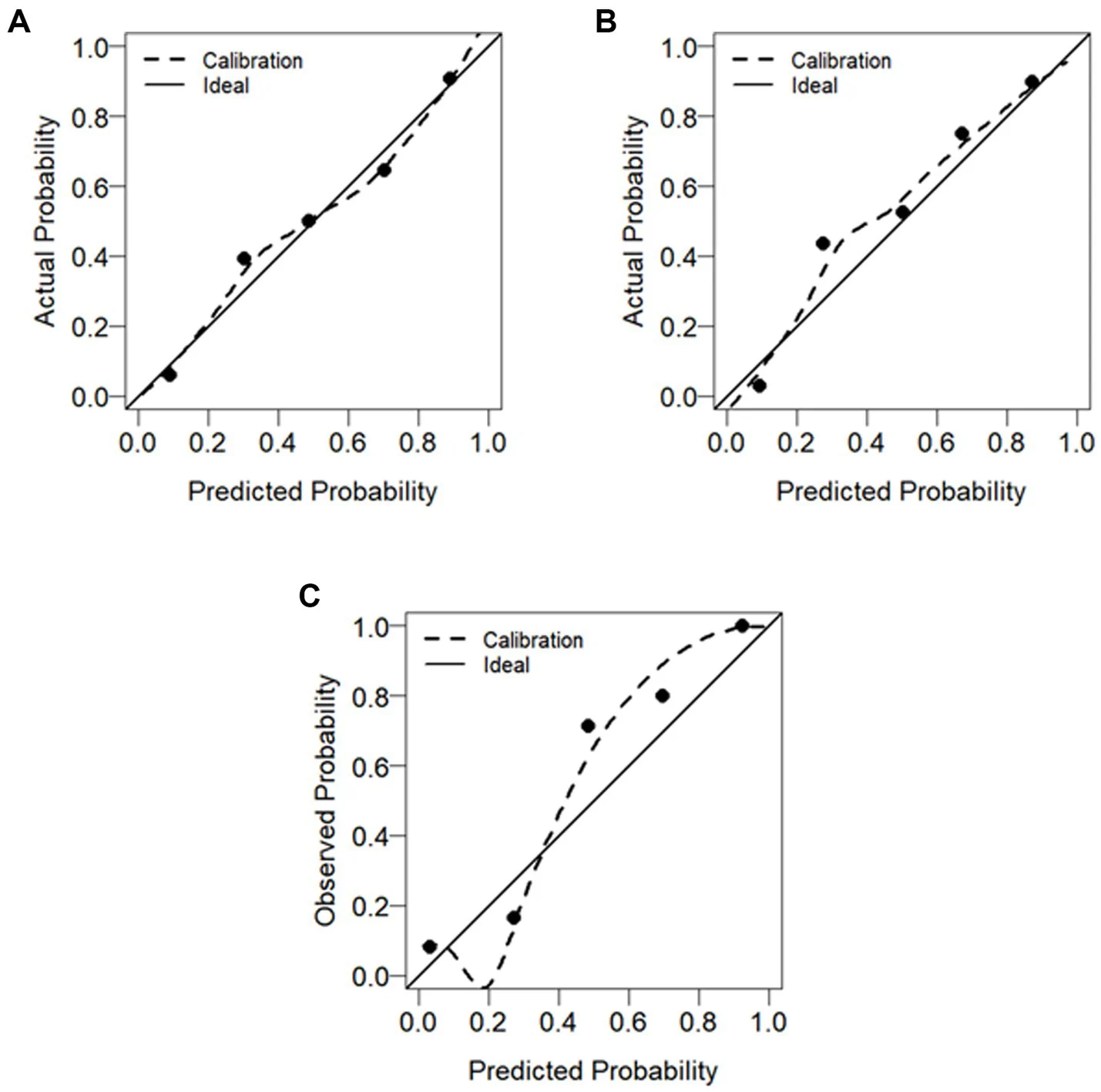
Calibration curves for the LR model. **(A)** Calibration curve for the training cohort; **(B)** Calibration curve for the internal validation cohort; **(C)** Calibration curve for the prospective validation cohort.

Calibration curves for the LR model in the training set and internal validation set closely approximated the ideal diagonal (Figures 4A,B). The Hosmer–Lemeshow test confirmed good calibration for the internal validation set (*χ^2^* = 2.291, *p* = 0.971). For the SVM + LR stacked ensemble, calibration in the training and internal validation sets was likewise satisfactory (Supplementary Figures S2A,B), with the Hosmer–Lemeshow test showing good fit in the internal validation set (*χ^2^* = 6.773, *p* = 0.561).

In the prospective validation cohort, both models showed evidence of miscalibration (Figure 4C; Supplementary Figure S2C). For the LR model, the Hosmer–Lemeshow test indicated significant deviation from ideal calibration (*χ^2^* = 124.461, *p* < 0.001). The prospective calibration slope was 0.7621, with a Brier score of 0.098. In addition, bootstrap validation performed in the development cohort showed a calibration slope of 0.7659 (95% CI: 0.4324–1.3843), suggesting moderate calibration stability after optimism correction.

The SVM + LR stacked ensemble showed a comparable calibration pattern in the prospective cohort, with Hosmer–Lemeshow *p* < 0.001, calibration slope = 1.408, calibration intercept = 0.620 (*p* = 0.155), and Brier score = 0.098. The model also exhibited modest underprediction of overall risk: 30 depressive events were observed versus 25.79 expected events, yielding an observed-to-expected (O:E) ratio of 1.163. Although the ensemble model exhibited a contrasting pattern of miscalibration (slope = 1.408, indicating underdispersion) compared with the LR model (slope = 0.762, indicating overdispersion), this did not translate into improved overall calibration performance, as both models demonstrated similar Brier scores and comparable calibration limitations. To evaluate whether the observed prospective miscalibration could be adjusted *post hoc*, we performed an exploratory logistic recalibration on the final LR model using the prospective cohort. The recalibration transformation was logit(*p*_cal_) = 0.0718 + 0.7048 × logit(*p_raw_*). The recalibrated model achieved perfect calibration within this dataset (intercept ≈ 0, slope = 1), shown in Supplementary Figure S4. Because recalibration and evaluation were performed on the same prospective cohort, these results are exploratory and hypothesis-generating; these recalibrated parameters require independent external validation before clinical application.

#### 3.4.2 Clinical utility validation

**Figure 5:**
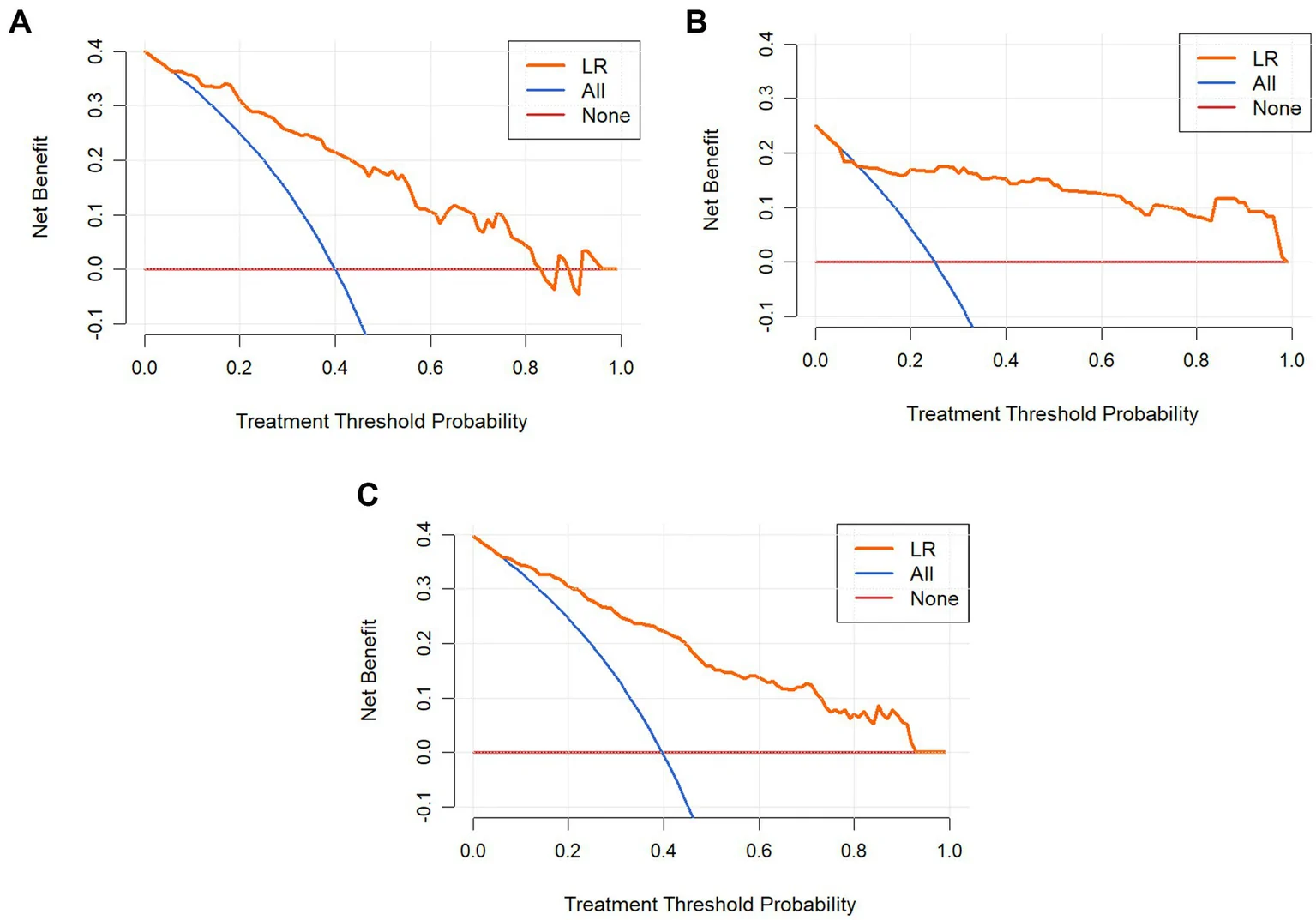
Decision curve analysis for the LR model. **(A)** DCA for the training cohort; **(B)** DCA for the internal validation cohort; **(C)** DCA for the prospective validation cohort.

The clinical utility of the LR model was evaluated via decision curve analysis (DCA) across the training, internal validation, and prospective validation sets (Figures 5A–C). In all three cohorts, the LR model provided stable positive net benefit above the “treat all” and “treat none” reference lines across a broad range of threshold probabilities. Particularly within the clinically relevant interval of 0.1–0.5, the model maintained a clear separation from both extreme strategies, supporting its utility for risk-stratified decision-making.

For direct head-to-head comparison, the SVM + LR stacked ensemble was evaluated in parallel (Supplementary Figures S3A–C). Visual inspection revealed that the net-benefit curves of the two models were virtually superimposable across all datasets. Across most threshold probabilities, the absolute difference in net benefit remained negligible, and neither model consistently dominated the other. These results confirm that the added algorithmic complexity of stacking did not translate into measurable incremental clinical net benefit.

Taken together with the marginally better calibration profile of the LR model in prospective validation, the absence of detectable DCA gain for the stacked ensemble supported the selection of the simpler, more interpretable logistic regression as the definitive clinical prediction tool.

A threshold sensitivity analysis across probability cutoffs of 0.10–0.50 in the prospective validation cohort confirmed that the 0.30 threshold provided a favorable balance between sensitivity, specificity, and net benefit (Supplementary Table S8). Detailed performance metrics and threshold selection rationale are provided in the supplementary analysis.

#### 3.4.3 Model interpretability analysis

**Figure 6:**
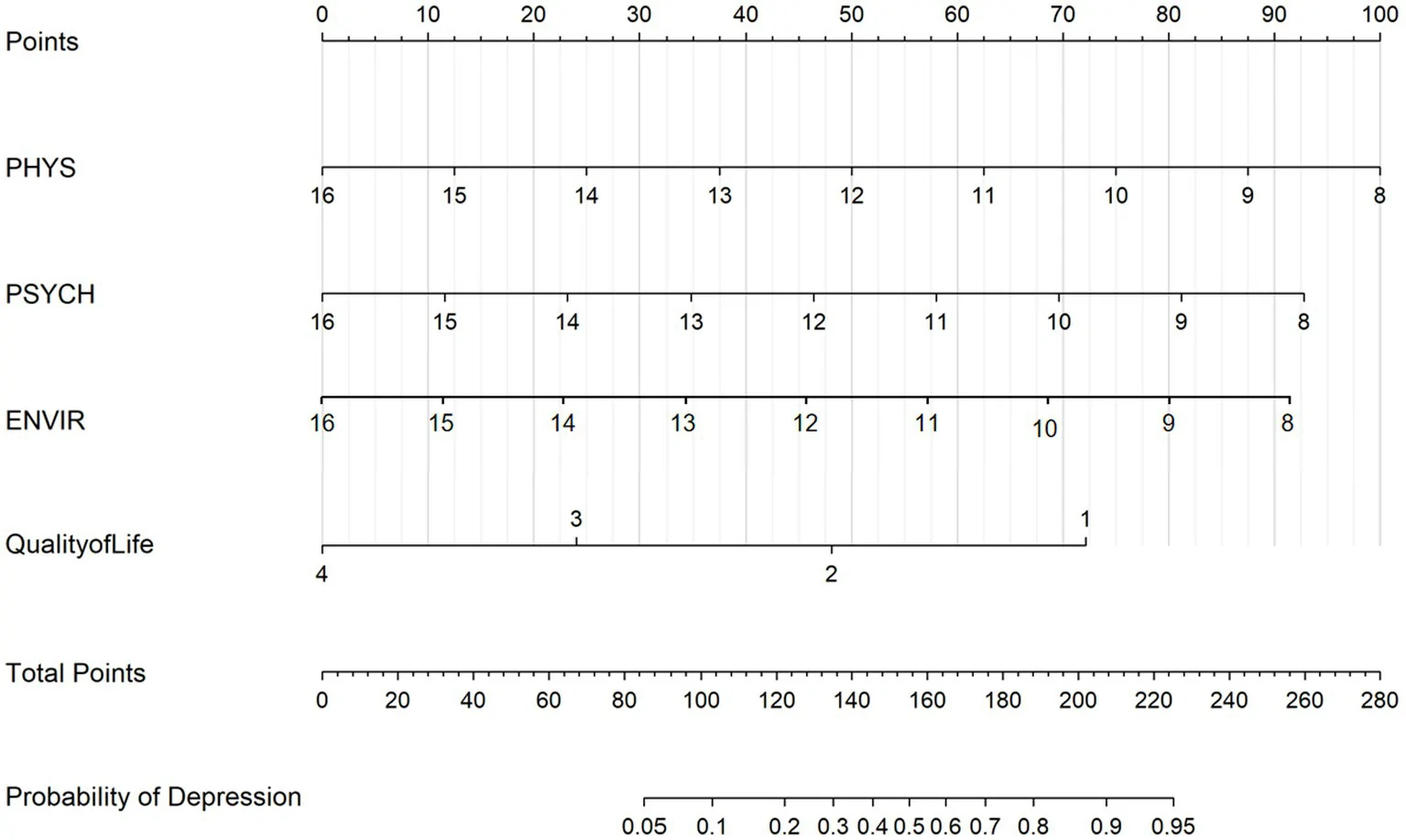
Nomogram for predicting depression risk in patients with AECOPD based on the final logistic regression model. PHYS, Physical function; PSYCH, Mental health; ENVIR, Environmental support. To use the nomogram, locate each patient’s value on the respective predictor axis, draw upward to determine the corresponding points, sum the points across all four predictors, and project the total score downward to obtain the predicted probability of depression.

To enhance clinical usability and transparency, a nomogram was constructed based on the final logistic regression model (Figure 6). The nomogram translates the four independent predictors—PHYS, PSYCH, ENVIR, and quality of life—into an intuitive, graphical scoring system. Each predictor axis is scaled according to its multivariable regression coefficient, allowing clinicians to assign points based on individual patient values. The accumulated total score is then mapped to a predicted probability of depression, ranging from 0.05 to 0.95.

As shown in Figure 6, PHYS, PSYCH, and ENVIR each span a comparable point range (approximately 0–100 points), indicating similar contributions to the composite risk estimate. Quality of life, scored ordinally from 1 to 5, exhibits a moderately narrower but clinically meaningful weight. Consistent with the negative coefficients observed in LASSO and logistic regression analyses, lower scores on each dimension (reflecting worse physical function, mental health, environmental support, and overall quality of life) correspond to higher assigned points and, consequently, higher predicted depression probabilities.

This visual decision-aid converts the multivariable model into a bedside calculation tool that does not require computational software, thereby supporting rapid risk stratification and individualized intervention planning in clinical practice.

#### 3.4.4 Model coefficient stability, multicollinearity assessment, and influencing factor analysis

As shown in Table 1, significant differences were observed between the retrospective and prospective cohorts in the four core predictive features (PHYS, PSYCH, ENVIR, quality of life) as well as in smoking duration and inflammation/coagulation indicators such as fibrinogen (FIB) (all *p* < 0.05). To determine whether these baseline differences affected the core predictive mechanisms of the final LR model, further analyses were conducted, including feature correlation analysis, multicollinearity diagnostics, ridge regression adjustment, and stratified analysis of key features. The results are as follows:

##### 3.4.4.1 Comparison of Core feature correlations

The correlation matrix of core features in the two cohorts showed that in the retrospective cohort, all feature correlation coefficients were below 0.63, with the lowest correlation observed between ENVIR and Quality of Life (*r* = 0.279) and the highest between PHYS and PSYCH (*r* = 0.624). In the prospective cohort, correlations among features were significantly stronger (*r* = 0.631–0.846), with the largest increase between PSYCH and ENVIR (Δ = 0.530) and the second largest between ENVIR and Quality of Life (Δ = 0.498), indicating stronger correlations among predictors and increased shared variance in the prospective cohort (Table 5).

**Table 5:** Comparison of correlation coefficients between core features in retrospective and prospective cohorts.

Feature Pair	Retrospective cohort, *r*	Prospective cohort, *r*	Δ*r*
PHYS vs. PSYCH	0.624	0.762	+0.138
PHYS vs. ENVIR	0.331	0.739	+0.408
PHYS vs. Quality of Life	0.341	0.631	+0.290
PSYCH vs. ENVIR	0.317	0.846	+0.530
PSYCH vs. Quality of Life	0.423	0.737	+0.314
ENVIR vs. Quality of Life	0.279	0.776	+0.498

*r* = Pearson correlation coefficient, only pairwise correlation results of 4 core predictive features are shown, all correlation coefficients are statistically significant (*p* < 0.05). Data source: Retrospective/prospective cohort analysis of this study.

##### 3.4.4.2 Multicollinearity diagnostics

In the retrospective cohort, the variance inflation factor (*VIF*) values of the four core features were all below 1.2 (mean *VIF* = 1.10), indicating no multicollinearity. In the prospective cohort, *VIF* values ranged from 2.64 to 4.61, with ENVIR having the highest *VIF* (4.61), and PSYCH and quality of life having *VIF*s of 3.43 and 3.93, respectively, suggesting moderate multicollinearity but not reaching commonly used thresholds for severe multicollinearity (*VIF* > 5 or > 10) (Table 6).

**Table 6:** Comparison of *VIF* values of core features between retrospective and prospective cohorts.

Feature Name	Retrospective cohort *VIF* value	Prospective cohort *VIF* value
PHYS	1.16	2.64
PSYCH	1.15	3.43
ENVIR	1.05	4.61
Quality of life	1.03	3.93
Mean *VIF* value	1.10	3.65

*VIF*, Variance Inflation Factor, Judgment criteria: *VIF*<3 indicates no collinearity, 3 ≤ *VIF*<10 indicates moderate collinearity, and *VIF*≥10 indicates severe collinearity. Data source: Retrospective/prospective cohort analysis of this study.

##### 3.4.4.3 Ridge regression adjustment results

After adjusting for multicollinearity using ridge regression (*λ* = 0.01), the coefficients of PHYS, PSYCH, and ENVIR remained negative in both cohorts, aligning with the direction observed in the LASSO selection. The coefficient of quality of life exhibited a complete directional reversal, changing from negative (−0.9107) in the retrospective cohort to positive (1.0022) in the prospective cohort (Table 7). This reversal was observed only in multivariable adjusted models and was not reproduced in univariate analyses or stratified depression prevalence analyses. This reversal is likely attributable to strengthened inter-predictor correlations and moderate multicollinearity observed in the prospective cohort (Tables 5, 6), rather than a true reversal of the underlying quality-of-life–depression association.

**Table 7:** Stability comparison of ridge regression coefficients.

Variable	Retrospective cohort ridge regression	Prospective cohort ridge regression	Direction
Intercept	15.7062	13.2751	—
PHYS	−0.4812	−0.3952	Negative, stable
PSYCH	−0.4618	−0.8315	Negative, stable
ENVIR	−0.1924	−0.1457	Negative, stable
Quality of Life	−0.9107	1.0022	Reversed

Data were directly extracted from the ridge regression output (λ = 0.01, determined by cross-validation), Stability criterion: Consistent coefficient direction = “Stable,” opposite direction = “Unstable.” Flipped” indicates a change from negative (retrospective) to positive (prospective). The “—” for the intercept denotes that directionality was not assessed for the intercept term, as it does not represent a predictor effect. Data source: Retrospective/prospective cohort analysis of this study.

##### 3.4.4.4 Stratified depression rate analysis by quality of life

After stratifying by quality of life scores, although baseline differences were observed between the two cohorts in core feature scores, smoking intervention, and inflammation management, the unadjusted association between quality of life score and depression prevalence remained consistent across cohorts. In the retrospective cohort, the subgroup with a quality of life score of 2 had the highest depression rate (70.6%), with a decreasing depression rate as the score increased. In the prospective cohort, there was no subgroup with a score of 1, but an additional subgroup with a score of 5 was included. In this cohort, the subgroup with a quality of life score of 2 had the highest depression rate (77.8%), higher than the same subgroup in the retrospective cohort. In both cohorts, the simple correlation coefficients between quality of life and depression were negative (retrospective cohort: *r* = −0.390, prospective cohort: *r* = −0.354), which is consistent with the results from LASSO regression and the univariate direction, suggesting that the predictive value of this feature is stable at the bivariate level despite multivariable coefficient instability (Table 8).

**Table 8:** Comparison of depression rates stratified by quality of life score.

Quality of life score	Retrospective cohort (*n* = 304)			Prospective cohort (*n* = 120)		
Sample size (*n*)	Depressive cases (*n*)	Sample size (*n*)	Depression rate (%)	Depressive cases (*n*)	Sample size (*n*)	Depression rate (%)
1	1	0	0.0	0	0	0
2	44	30	68.2	9	7	77.8
3	174	81	46.6	61	18	29.5
4	85	10	11.8	39	4	10.3
5	0	0	0	11	1	9.1

Data were derived from the retrospective and prospective cohort analyses of this study.

##### 3.4.4.5 Univariate feature-outcome associations across cohorts

To assess whether the direction of association between each core predictor and depression was stable between the retrospective and prospective cohorts, univariate logistic regression was performed for each feature. All four features (PHYS, PSYCH, ENVIR, quality of life) exhibited negative coefficients in both cohorts, supporting consistent univariate associations across cohorts. This confirms that higher scores on these domains are consistently associated with lower depression risk at the unadjusted level, though multivariable coefficient behavior may differ due to predictor intercorrelations.

Overall, these analyses indicate that the predictive direction of individual features remained consistent across cohorts at the univariate level. However, stronger correlations among predictors in the prospective cohort (Table 5) introduced multivariable coefficient instability, particularly for Quality of Life, which exhibited a complete directional reversal in the prospective multivariable models (Table 7). This reversal is interpreted as a multivariable estimation artifact likely related to altered predictor correlation structure and moderate multicollinearity (mean *VIF* = 3.65) in the prospective cohort, rather than a true change in the underlying negative association between quality of life and depression risk.

#### 3.4.5 Sensitivity analysis addressing potential construct overlap

To address potential construct overlap between the WHOQOL-BREF PSYCH domain and the PHQ-9 outcome measure, we evaluated whether model discrimination was substantially driven by this psychological dimension. Although the WHOQOL-BREF PSYCH domain captures broader mental health-related quality of life (including positive affect, self-esteem, body image, and cognitive function) rather than depressive symptom frequency per se, we acknowledge that it contains items conceptually related to low mood and psychological distress.

Exclusion of the PSYCH domain resulted in a modest decrease in prospective discrimination (*AUC* 0.848, 95% CI: 0.746–0.949) compared with the primary four-feature model (*AUC* 0.866, 95% CI: 0.772–0.960). Further exclusion of the Quality of Life domain yielded comparable performance (*AUC* 0.852, 95% CI: 0.752–0.952). Notably, the PSYCH-only model showed substantially lower discrimination, with prospective *AUC* of 0.785 (95% CI: 0.700–0.860), indicating that no single domain-psychological or otherwise-dominates prediction. These findings demonstrate that the model’s predictive performance is not exclusively dependent on potentially overlapping psychological constructs and remains clinically useful even when PSYCH is omitted (Table 9).

**Table 9:** Univariate logistic regression coefficients for core features in retrospective and prospective cohorts.

Feature	Retrospective coefficient	Prospective coefficient
PHYS	−0.815	−1.075
PSYCH	−1.015	−1.142
ENVIR	−0.372	−0.994
Quality of life	−1.313	−1.368

This suggests that the PHQ-9 and WHOQOL-BREF PSYCH, while correlated, measure distinct constructs: the PHQ-9 quantifies depressive symptom frequency over the preceding 2 weeks, whereas PSYCH reflects a broader, health-related quality-of-life framework. Nevertheless, in settings where minimization of any conceptual overlap is prioritized, the three-feature model (PHYS, ENVIR, quality of life) offers a viable alternative with only modest sacrifice in discrimination.

## 4 Discussion

This study used a retrospective development cohort and a temporal prospective validation cohort to develop and evaluate a prediction model for identifying concurrent depressive symptoms among hospitalized AECOPD patients. LASSO regression was applied for feature selection, and four core predictors (PHYS, PSYCH, ENVIR, and quality of life) were subsequently incorporated into an interpretable logistic regression model. Although several machine-learning algorithms, including an SVM + LR stacking model, were explored, the final clinical model was the logistic regression model because the ensemble approach did not provide significant improvement in discrimination, calibration, or clinical utility.

The final LR model demonstrated stable discrimination across cohorts, with *AUC* values of 0.866, 0.862, and 0.866 in the training, internal validation, and prospective validation cohorts, respectively. In the prospective cohort, the model achieved an accuracy of 88.3%, specificity of 91.1%, and Brier score of 0.098. Calibration assessment, decision curve analysis, and the developed nomogram demonstrated the potential clinical applicability of the model. The identified predictors provide an interpretable framework for early risk stratification and targeted psychological assessment in AECOPD patients.

### 4.1 Clinical significance and mechanistic interpretation of core predictive features

The four core features identified in this study were independent predictors of depression risk in AECOPD patients. The negative LASSO coefficients (−0.074711, −0.038484, −0.003864, −0.066359) and logistic regression coefficients consistently indicated that lower scores were associated with higher depression risk. This observation aligns with the data trends in this study and is supported by previous research.

PHYS reflects impaired physical function in AECOPD patients, associated with dyspnea and activity limitation (7, 9, 13). Although PHYS scores differed significantly between retrospective and prospective cohorts (*p* < 0.001), the negative predictive effect of this feature was consistent, confirming the intrinsic association between physical function and depression risk. Previous studies have shown that symptom burden is strongly related to depression in COPD patients, with more severe symptoms corresponding to higher risk. Research also suggests that symptom burden exerts a stronger impact on depression than lung function measures, supporting the clinical relevance of the PHYS dimension.

PSYCH represents patients’ emotional state and is closely related to depression risk (5). Despite significantly higher PSYCH scores in the prospective cohort compared with the retrospective cohort (*p* < 0.001), the regression coefficient remained directionally consistent, indicating a stable association between psychological status and depressive symptom risk. Previous studies have reported that negative emotions such as anxiety and low mood are common among COPD patients and significantly correlate with depression severity (14, 15). Moreover, interactions between psychological stress and inflammatory responses are thought to contribute to the development of mood disorders (16).

Although potential conceptual overlap may exist between psychologically related WHOQOL-BREF domains and PHQ-9 depressive symptoms, sensitivity analyses demonstrated that model discrimination remained acceptable after excluding the PSYCH domain (prospective validation *AUC* = 0.8476, 95% CI: 0.7462–0.9490) and after further exclusion of subjective quality-of-life information (prospective validation *AUC* = 0.8522, 95% CI: 0.7522–0.9522). These findings suggest that model performance was not exclusively driven by overlapping psychological constructs.

ENVIR reflects social support levels (9). In this study, ENVIR scores differed significantly between the two cohorts (*p* < 0.001), but after ridge regression correction for multicollinearity, its coefficient direction remained consistent with that in the retrospective cohort, confirming a stable negative association between social support and depression risk. Epidemiological studies have highlighted that COPD patients with insufficient social support are more likely to develop depressive symptoms (17–19), emphasizing the buffering role of the social environment in psychological regulation.

Quality of life represents an integrated measure of physical function, mental state, and social adaptation, enabling the model to overcome limitations of single dimension (7). Stratified analyses indicated significant differences in quality of life scores between cohorts (*p* < 0.001), with an additional subgroup scoring 5 in the prospective cohort (depression rate 9.1%). Nonetheless, in both cohorts, quality of life remained negatively correlated with depression at the univariate level (retrospective cohort *r* = −0.390, prospective cohort *r* = −0.354), supporting a stable negative underlying association despite multivariable coefficient instability in the prospective cohort. This cross-cohort inconsistency in the multivariable coefficient likely reflects the strengthened inter-predictor correlations and moderate multicollinearity observed in the prospective validation cohort (Tables 5, 6), not a true reversal of the clinical relationship. Prior research has shown that decreased quality of life is significantly associated with depressive symptoms in COPD patients, particularly during acute exacerbations (20, 21).

It is important to note that while the underlying negative association between quality of life and depression remained consistent across cohorts at the univariate level, the quality-of-life coefficient exhibited a complete directional reversal in the prospective multivariable ridge regression model. This multivariable instability likely reflects strengthened inter-predictor correlations and moderate multicollinearity in the prospective cohort (Tables 5, 6), underscoring the need for cautious interpretation of individual coefficient directions when predictor distributions shift across cohorts.

### 4.2 Model performance, interpretability, and clinical utility

The final LR model demonstrated favorable predictive performance while maintaining interpretability and clinical applicability. Although advanced machine-learning algorithms were evaluated, including SVM and SVM + LR stacking models, none showed statistically significant or clinically meaningful superiority over LR. The comparable discrimination among these approaches suggests that the relatively small number of selected predictors and their strong clinical interpretability may limit the additional value gained from more complex algorithms.

Logistic regression provides direct estimation of predictor effects and allows construction of an individual risk prediction nomogram. In this study, higher PHYS, PSYCH, ENVIR, and quality-of-life scores were consistently associated with lower depression risk, supporting the clinical interpretability of the model. Compared with more complex machine-learning approaches, the LR model offers advantages in transparency, reproducibility, and potential integration into routine clinical workflows.

Model evaluation using discrimination, calibration assessment, and decision curve analysis further supported its clinical relevance. However, the observed calibration deterioration during prospective validation highlights the need for external validation and potential recalibration before widespread clinical implementation.

### 4.3 Clinical translational value and practical application scenarios

To facilitate model interpretation and research reproducibility, a freely accessible web-based research prototype based on the final LR model was developed. The calculator provides individualized risk estimation using four routinely obtainable predictors and may assist clinicians in identifying patients requiring further psychological assessment.

In outpatient scenarios, clinicians can quickly assess the four core features, perform PHQ-9 evaluation, or refer high-risk patients to psychiatry, improving screening efficiency. In inpatient settings, model predictions can be incorporated into admission assessment systems, prompting early intervention for high-risk patients with low PHYS scores or limited social support, such as combining pulmonary rehabilitation to improve physical function, cognitive-behavioral therapy to regulate mental state, and family or community support networks, achieving multidimensional precise intervention.

The core features provide actionable targets for clinical intervention. For patients with low PHYS scores, noninvasive ventilation support or respiratory rehabilitation may be considered, for abnormal PSYCH scores, cognitive-behavioral therapy or pharmacological interventions may be applied, for low ENVIR scores, a coordinated support system involving family, community, and healthcare providers can be established, and comprehensive improvement in quality of life requires multidisciplinary collaboration integrating physiological, psychological, and social interventions. This screening-to-intervention framework may facilitate early identification and intervention, significantly improving overall patient outcomes.

The calculator provides relative risk estimates and is available at: https://zhouxinyue.shinyapps.io/aecopd_calculator_shiny/. The calculator is provided for research and demonstration purposes only and requires prospective usability testing, clinical workflow integration assessment, and comparative effectiveness evaluation before any clinical use. The model showed relatively better performance in patients with intermediate quality of life scores (quality of life = 3), whereas performance in other strata was less stable in the current dataset; therefore, outputs for these subgroups should be interpreted with caution.

### 4.4 Limitations and future research directions

First, the training sample size was adequate for development of the parsimonious logistic regression model but may have been insufficient for reliable exploration of complex machine-learning architectures; therefore, the machine-learning comparisons should be considered exploratory. In addition, the four core features differed significantly between the retrospective and prospective cohorts (Table 1), which limits the independence of the prospective validation. In particular, the quality-of-life coefficient exhibited a complete directional reversal in the prospective multivariable ridge regression model (from −0.9107 in the retrospective cohort to +1.0022 in the prospective cohort; Table 7). This reversal was observed only in multivariable adjusted models and was not reproduced in univariate analyses or stratified depression prevalence analyses (Tables 8, 10), indicating that it reflects multivariable estimation instability likely attributable to strengthened inter-predictor correlations and moderate multicollinearity in the prospective cohort (Tables 5, 6) rather than a true reversal of the underlying quality-of-life–depression association.

**Table 10:** Sensitivity analyses addressing potential construct overlap between WHOQOL-BREF domains and PHQ-9 depressive symptoms.

Model	Training *AUC* (95% CI)	Internal validation *AUC* (95% CI)	Prospective validation *AUC* (95% CI)
LR	0.8659 (0.8181–0.9137)	0.8624 (0.7885–0.9362)	0.8663 (0.7722–0.9604)
LR excluding PSYCH	0.8592 (0.8103–0.9082)	0.8419 (0.767–0.917)	0.8476 (0.7462–0.9490)
LR excluding PSYCH + Quality of Life	0.8400 (0.7880–0.8921)	0.8547 (0.7793–0.9300)	0.8522 (0.7522–0.9522)
PSYCH-only LR	0.7834 (0.7237–0.8432)	0.7245 (0.6236–0.8255)	0.7854 (0.70–0.86)

Variable compositions are as follows: Primary LR model includes PHYS, PSYCH, ENVIR, and Quality of Life; LR excluding PSYCH includes PHYS, ENVIR, and Quality of Life; LR excluding PSYCH + Quality of Life includes PHYS and ENVIR; PSYCH-only LR includes PSYCH only. PHYS, physical function domain; PSYCH, mental health domain; ENVIR, environmental support domain.

Prospective performance varied across quality of life strata. The model showed poor discriminative ability in patients with quality of life ≥ 4 (*AUC* = 0.6, 95% CI: 0.351–0.849), indicating unreliable predictions in this subgroup. In contrast, performance showed relatively better discrimination in patients with quality of life = 3 (*n* = 61, *AUC* = 0.938, 95% CI: 0.865–1.000), which accounted for approximately half of the prospective cohort. Therefore, the model may be more appropriate as a risk stratification tool for patients with intermediate quality of life rather than for precise probability estimation. Patients with quality of life ≤ 2 were underrepresented, limiting reliable evaluation in these subgroups. Further multicenter studies with larger and more balanced samples are needed to validate model performance across quality of life strata.

Second, in the prospective single-center validation, the final LR model showed evidence of calibration deterioration (calibration slope = 0.7621, Hosmer-Lemeshow *p* < 0.001), indicating overdispersion of predicted probabilities. The observed-to-expected ratio was 1.163, reflecting modest underprediction of overall event risk. This likely reflects differences in feature distributions, outcome prevalence, and clinical management between cohorts (Table 1). We performed an exploratory logistic recalibration on the same cohort (see Results 3.4.1) to illustrate potential calibration adjustment. This analysis showed improved apparent calibration within this dataset without loss of discrimination. However, as both recalibration and evaluation were performed on the same cohort, these results are exploratory and do not provide evidence of performance in independent populations. External validation and local recalibration that may be necessary are required before clinical application.

Third, this was a single-center study, with both retrospective and prospective data derived from the same institution. Therefore, the prospective validation does not demonstrate external generalizability. The reported prospective *AUC* of 0.866 (95% CI: 0.772–0.960) should be interpreted as temporal validation rather than external validation. Independent multicenter validation is required prior to broader clinical use.

Fourth, the model did not incorporate biomarker data (e.g., IL-6, TNF-*α*, fibrinogen), which may further improve predictive performance through multimodal integration. Future studies should explore the addition of inflammatory biomarkers to enhance model robustness.

Fifth, the study lacked longitudinal follow-up, limiting assessment of long-term outcomes and the potential impact of model-guided interventions. Prospective longitudinal and interventional studies are therefore warranted.

Sixth, direct comparison of questionnaire burden, completion time, missingness, and incremental predictive value against PHQ-9 screening alone was not performed. Therefore, the incremental clinical benefit of incorporating WHOQOL-BREF-derived information beyond PHQ-9 alone remains uncertain. Additionally, the 0.30 probability threshold was selected based on the training cohort and a clinical prioritization of sensitivity; locally derived thresholds may be preferable when implementing this model in populations with different depression prevalence or healthcare resource constraints.

Seventh, this study did not include a direct comparison between the model-assisted strategy and routine PHQ-9 screening. Therefore, whether the proposed approach improves screening efficiency, clinical outcomes, or resource utilization remains to be determined in future prospective comparative studies.

Of note, the PHQ-9 cut-off of ≥5 was selected to prioritize sensitivity for identifying patients with possible depressive symptoms in a screening-oriented setting, where symptom overlap and underdiagnosis are common. This threshold has also been applied in previous AECOPD studies (15). However, it yields a higher prevalence (39.8%) compared with the conventional PHQ-9 ≥ 10 threshold for major depressive disorder. Therefore, comparisons with other studies should consider differences in cut-off selection and clinical context. Sensitivity analysis using PHQ-9 ≥ 10 was precluded by insufficient event numbers in the development cohort (n = 3). The outcome definition in this study was based on PHQ-9 ≥ 5, representing at least mild depressive symptoms rather than moderate-to-severe depression. Therefore, comparisons with studies using higher PHQ-9 thresholds should consider differences in outcome phenotypes.

Overall, future research should focus on multicenter, longitudinal, and multimodal validation, including integration of inflammatory biomarkers and independent external datasets, to improve the robustness and generalizability of AECOPD depression prediction models.

## 5 Conclusion

Based on the four core features PHYS, PSYCH, ENVIR, and quality of life, this study developed and temporally validated an interpretable logistic regression prediction model. The final recommended clinical tool was an interpretable logistic regression model to identify concurrent depressive symptoms during hospitalization in AECOPD patients. The model demonstrated good discrimination and clinical utility; however, prospective calibration assessment revealed deterioration, highlighting the need for external validation and potential local recalibration before clinical implementation. The nomogram and regression coefficients provided an interpretable representation of individual risk estimation. This model can serve as an auxiliary tool for early identification of high-risk patients, supporting the development of individualized intervention strategies and potentially supporting subsequent psychological assessment and individualized intervention planning. Future studies incorporating multicenter validation, biomarker integration, intervention effectiveness assessment, and clinician acceptability evaluation may further optimize model performance and promote its broad clinical application.

## Data Availability

The raw data supporting the conclusions of this study cannot be publicly shared due to participant‑privacy‑related ethical restrictions. Anonymized aggregated data may be available from the corresponding author upon reasonable request.

## Glossary

AECOPD: Acute Exacerbation of Chronic Obstructive Pulmonary Disease
COPD: Chronic Obstructive Pulmonary Disease
LASSO: Least Absolute Shrinkage and Selection Operator
SVM: Support Vector Machine
LR: Logistic Regression
DT: Decision Tree
PLS: Partial Least Squares
GBM: Gradient Boosting Machine
NB: Naive Bayes
RF: Random Forest
SVC: Support Vector Classifier
XGB: Extreme Gradient Boosting
KNN: K-Nearest Neighbors
*AUC*: Area Under the Curve
PHYS: Physical Function
PSYCH: Mental Health
ENVIR: Environmental Support
DCA: Decision Curve Analysis
PHQ-9: Patient Health Questionnaire-9
SIS: Social Impact Scale
mMRC: Modified Medical Research Council
GOLD: Global Initiative for Chronic Obstructive Lung Disease
FEV1/FVC: Forced Expiratory Volume in 1 Second/Forced Vital Capacity
BMI: Body Mass Index
ROC: Receiver Operating Characteristic
CI: Confidence Interval
WBC: White Blood Cell Count
Neu: Neutrophil Percentage
NEU: Absolute Neutrophil Count
RBC: Red Blood Cell Count
Hb: Hemoglobin
PLT: Platelet Count
PT: Prothrombin Time
PT%: Prothrombin Time Activity
APTT: Activated Partial Thromboplastin Time
FIB: Fibrinogen
TT: Thrombin Time
D-Dimer: D-Dimer
ALT: Alanine Aminotransferase
AST: Aspartate Aminotransferase
TBIL: Total Bilirubin
TP: Total Protein
ALB: Albumin
eGFR: Estimated Glomerular Filtration Rate
GLU: Blood Glucose
K^+^: Potassium Ion
Na^+^: Sodium Ion
SOCIL: Social Support Score
IL-6: Interleukin-6
TNF-α: Tumor Necrosis Factor-α
