# Supplementary material for: Development and temporal prospective validation of an interpretable machine learning model for identifying depressive symptoms in patients with acute exacerbation of chronic obstructive pulmonary disease
Source: Front Public Health. 2026 Sep 8;14:1850011. doi: 10.3389/fpubh.2026.1850011 (PMC13593758; doi:10.3389/fpubh.2026.1850011)
Supplement: Supplementary file 1 [file Data_Sheet_1.pdf]

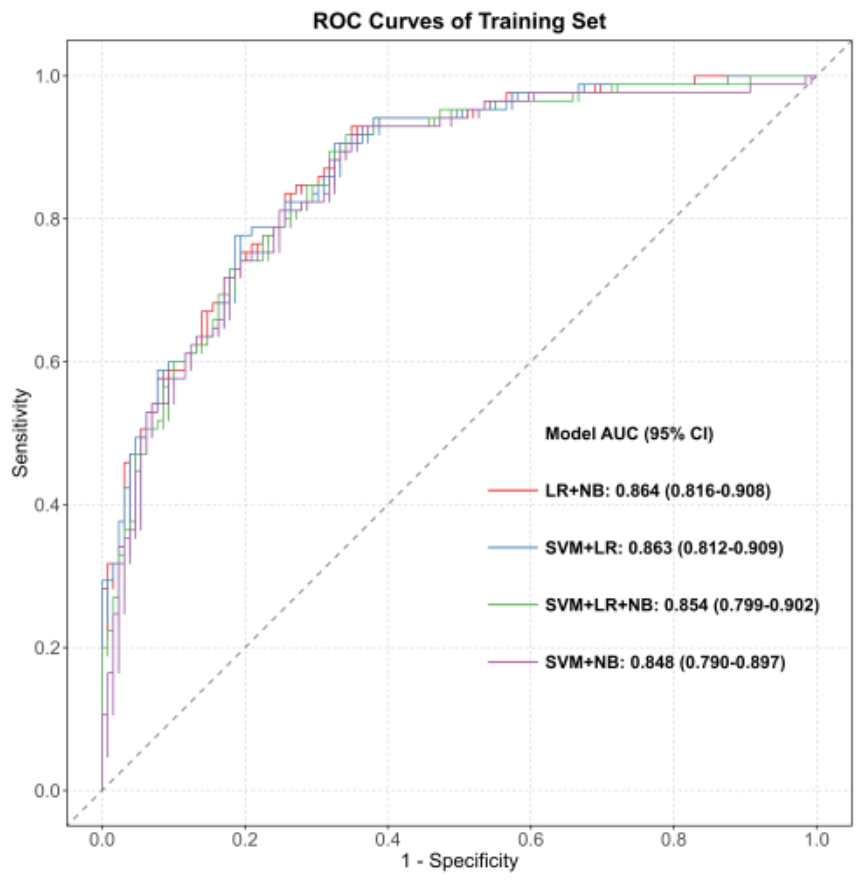

Figure A

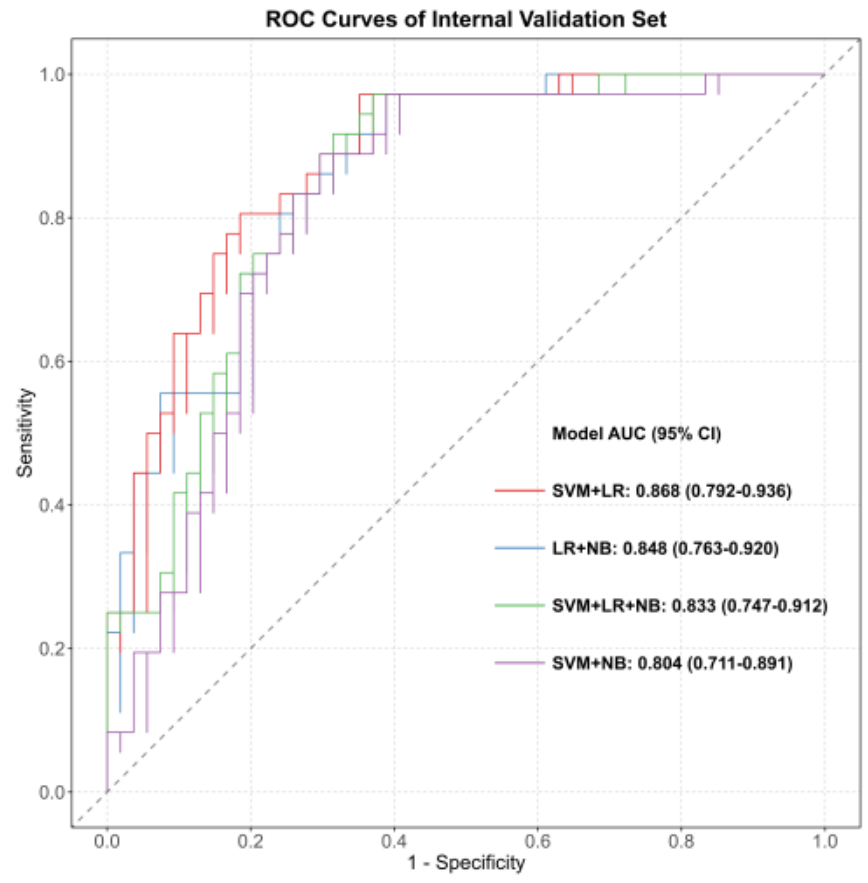

Figure B

Supplementary Figure S1. (A) ROC curves of stacking-ensemble models in the training set; (B) ROC curves of stacking-ensemble models in the internal validation set.

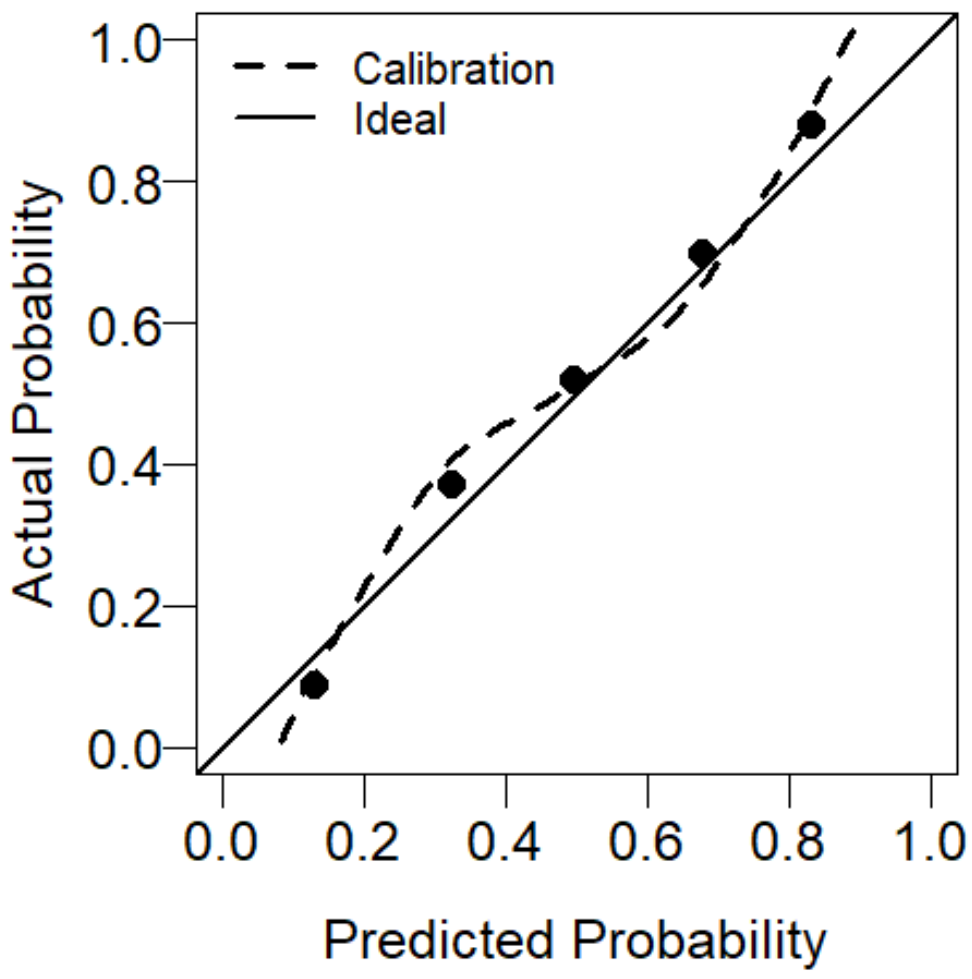

Figure A

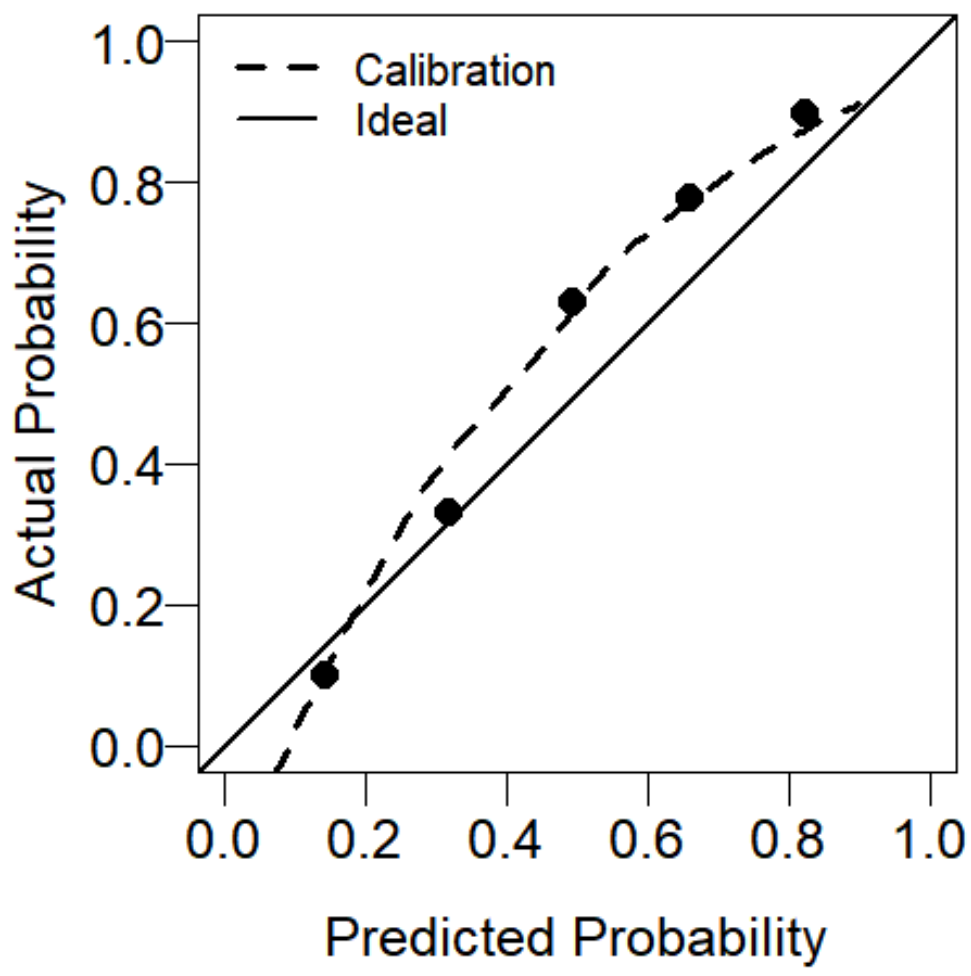

Figure B

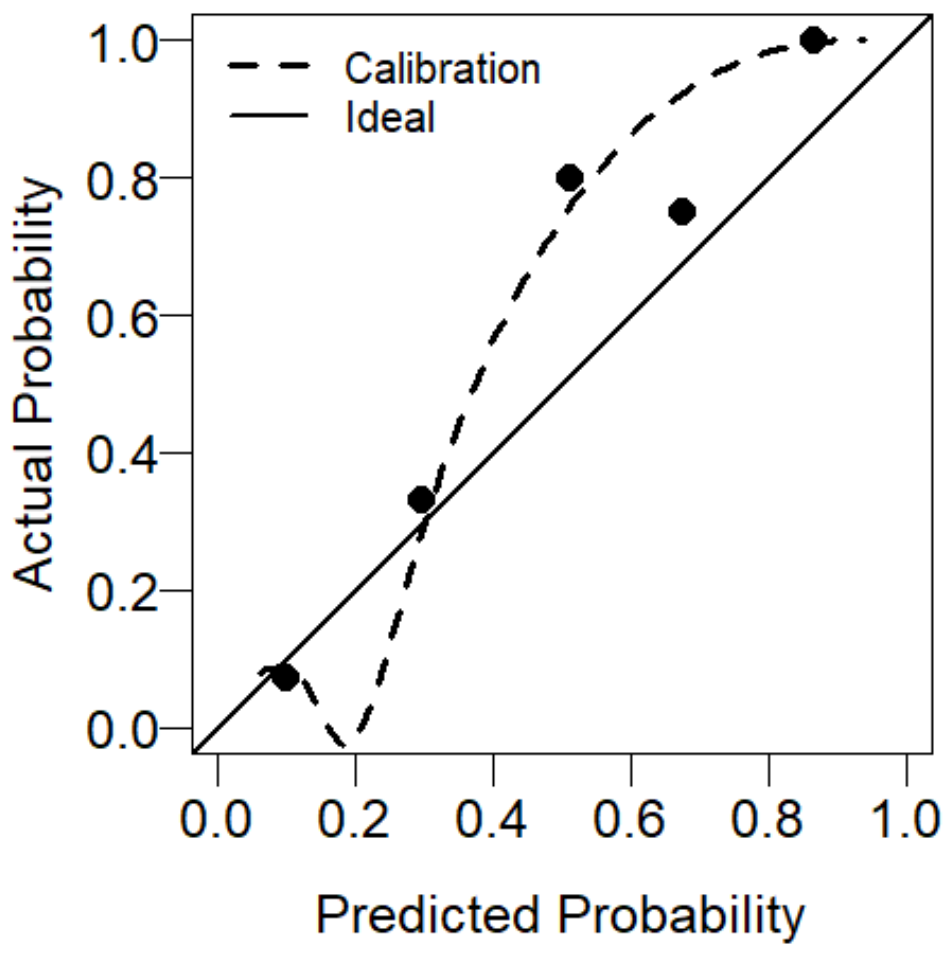

Figure C

Supplementary Figure S2. Calibration curves for the SVM+LR stacked ensemble.(A)

Calibration curve for the training cohort, (B) Calibration curve for the internal validation cohort.(C) Calibration curve for the prospective validation Cohort.

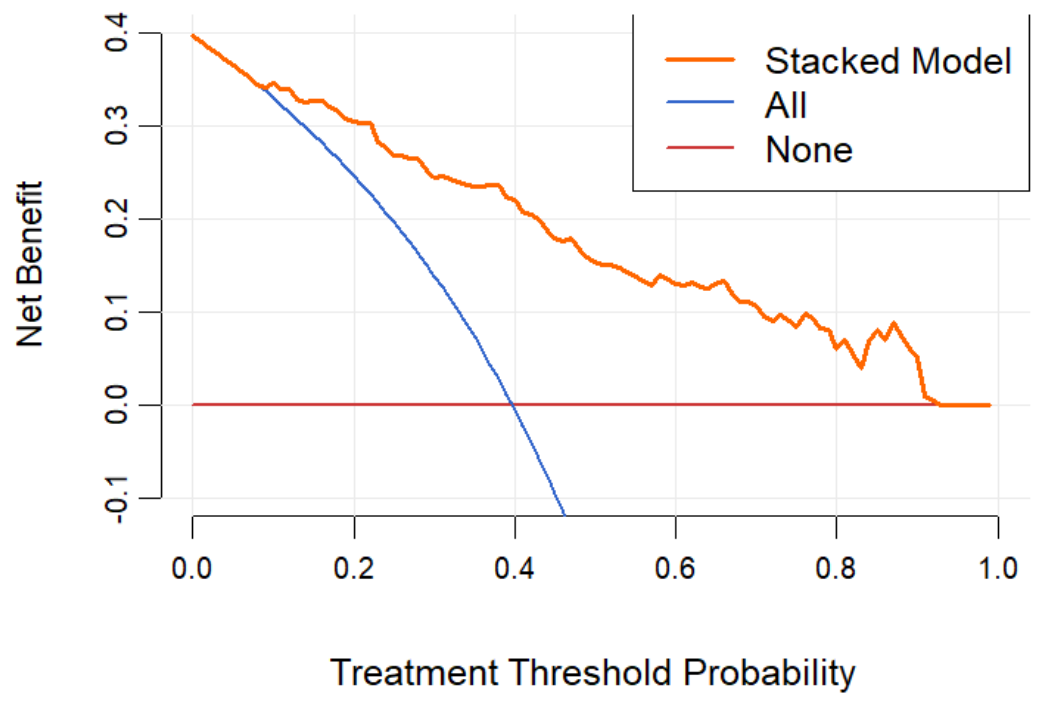

Figure A

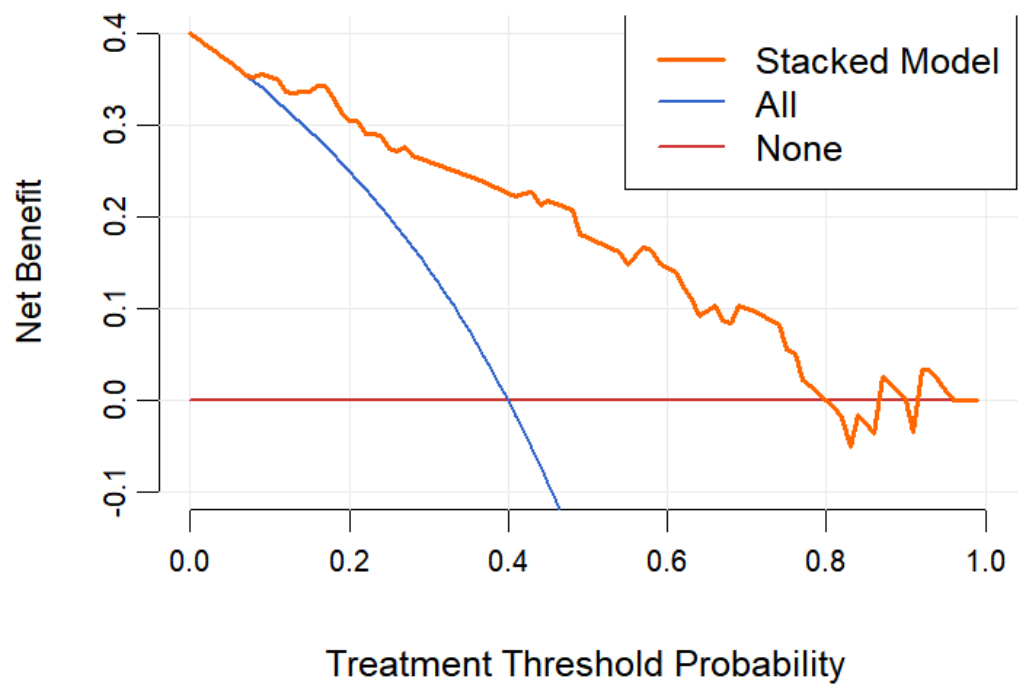

Figure B

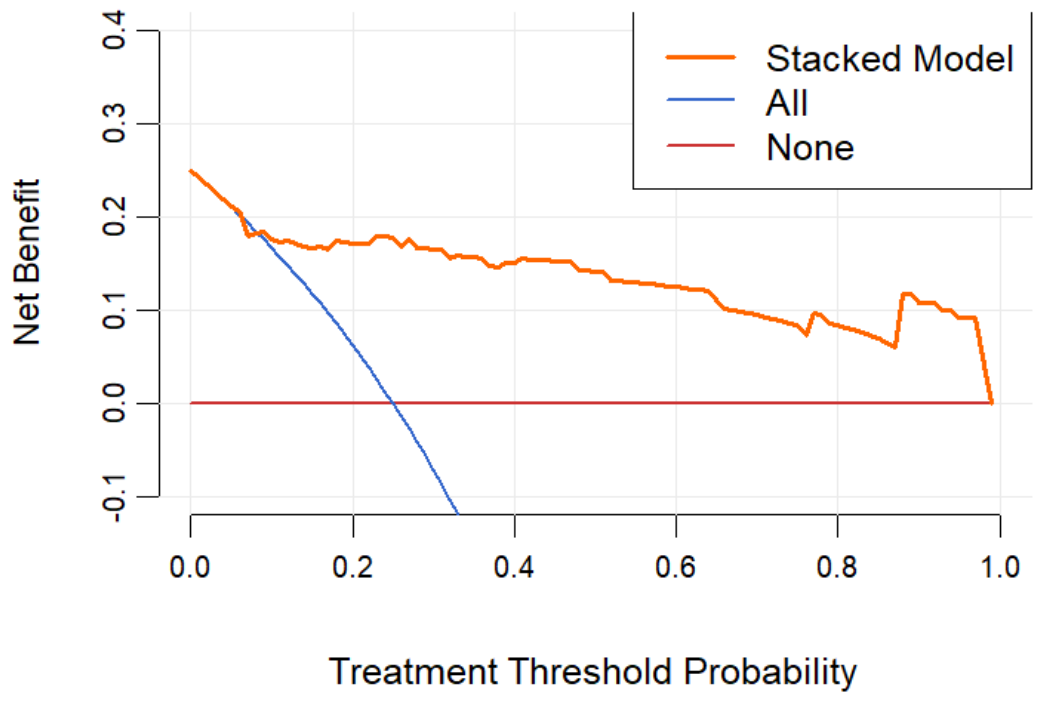

Figure C

Supplementary Figure S3. Decision curve analysis for the SVM+LR stacked ensemble model. (A) DCA for the training cohort, (B) DCA for the internal validation cohort. (C)DCA for the prospective validation cohort.

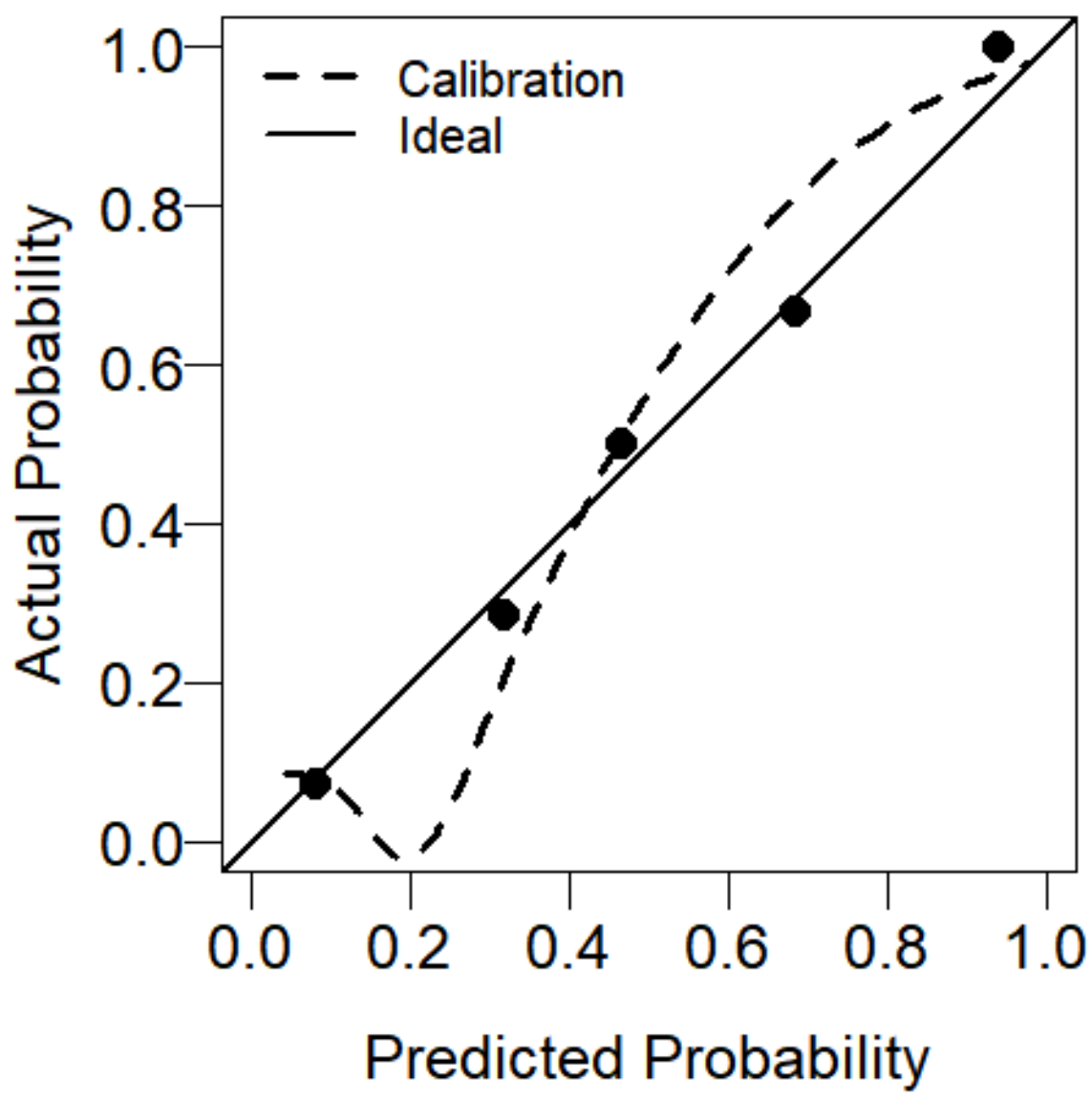

Supplementary Figure S4. Calibration curve of the final logistic-regression model after exploratory logistic recalibration in the prospective validation cohort. Recalibration was

performed and assessed within the same prospective cohort; these results are exploratory for reference.
