# Supplementary material for: Development and temporal prospective validation of an interpretable machine learning model for identifying depressive symptoms in patients with acute exacerbation of chronic obstructive pulmonary disease
Source: Front Public Health. 2026 Sep 8;14:1850011. doi: 10.3389/fpubh.2026.1850011 (PMC13593758; doi:10.3389/fpubh.2026.1850011)
Supplement: Supplementary file 2 [file Table_1.DOCX]

**Supplementary Table S1.** Software environment and R package versions

| caret | 7.0-1 |
| --- | --- |
| glmnet | 4.1-10 |
| e1071 | 1.7-16 |
| naivebayes | 1.0.0 |
| randomForest | 4.7-1.2 |
| xgboost | 1.7.11.1 |
| gbm | 2.2.2 |
| pls | 2.8-5 |
| pROC | 1.19.0.1 |
| dplyr | 1.1.4 |
| caret | 7.0-1 |

**Note:** The complete R sessionInfo() output is provided in Supplementary File S1 to ensure full computational reproducibility.
