# Supplementary material for: Development and temporal prospective validation of an interpretable machine learning model for identifying depressive symptoms in patients with acute exacerbation of chronic obstructive pulmonary disease
Source: Front Public Health. 2026 Sep 8;14:1850011. doi: 10.3389/fpubh.2026.1850011 (PMC13593758; doi:10.3389/fpubh.2026.1850011)
Supplement: Supplementary file 3 [file Table_2.DOCX]

**Supplementary Table S2.** Baseline machine-learning model specifications: kernels, tuning grids, and selected hyperparameters

| Model | caret Method | Kernel/Key Parameters | Tuning Grid | Final Selected Values |
| --- | --- | --- | --- | --- |
| LR | glm | Binomial logit | Default (none) | Maximum likelihood estimation |
| SVM | svmLinear | Linear kernel | C: {0.1, 0.5, 1, 2, 5} | C = 1 |
| SVC | svmRadial | RBF kernel | C: {0.1, 0.5, 1, 2, 5};  sigma: via kernlab::sigest | C = 0.25,  sigma = 0.264 |
| NB | naive_bayes | Gaussian/kernel density | fL: {0, 1, 2};  usekernel:{FALSE, TRUE};  adjust: {0.5, 1, 2} | fL = 0,  usekernel = FALSE, adjust = 1 |
| RF | rf | Bagged trees | mtry: {1, 2, 3} | mtry = 2 |
| GBM | gbm | Gradient boosting | n.trees:{100,200}; interaction.depth: {1, 2, 3}; shrinkage: {0.1, 0.01} | n.trees=100, interaction.depth= 2, shrinkage = 0.1 |
| XGBoost | xgbTree | Extreme gradient boosting | nrounds:{100,150}; max_depth: {1, 2, 3};  eta: {0.3, 0.4};  subsample:{0.8,1}; colsample_bytree:{0.8, 1} | nrounds=150, max_depth = 1,  eta = 0.4 |
| DT | rpart | CART | cp: caret default grid | cp = 0.01176 |
| PLS | pls | Partial least squares | ncomp: {1, 2, 3} | ncomp = 3 |
| KNN | knn | K-nearest neighbours | k: {3, 5, 7, 9} | k = 7 |

**Note:** For algorithms with tunable parameters, optimization was conducted exclusively through internal resampling within the training set. No validation data were used for parameter selection. cp = complexity parameter; mtry = number of variables randomly sampled as candidates at each split; ncomp = number of components.
