# Supplementary material for: Development and temporal prospective validation of an interpretable machine learning model for identifying depressive symptoms in patients with acute exacerbation of chronic obstructive pulmonary disease
Source: Front Public Health. 2026 Sep 8;14:1850011. doi: 10.3389/fpubh.2026.1850011 (PMC13593758; doi:10.3389/fpubh.2026.1850011)
Supplement: Supplementary file 4 [file Table_3.DOCX]

**Supplementary Table S3.** Meta-logistic regression coefficients of the final SVM+LR stacking ensemble

| Parameter | Coefficient | Interpretation |
| --- | --- | --- |
| Intercept  (β₀) | -3.1234 | Baseline log-odds when both base-model probabilities are zero |
| LR probability coefficient ( β₁) | 5.6789 | Log-odds change per unit increase in LR predicted probability |
| SVM probability coefficient (β₂) | 0.8765 | Log-odds change per unit increase in SVM predicted probability |
