# Supplementary material for: Development and temporal prospective validation of an interpretable machine learning model for identifying depressive symptoms in patients with acute exacerbation of chronic obstructive pulmonary disease
Source: Front Public Health. 2026 Sep 8;14:1850011. doi: 10.3389/fpubh.2026.1850011 (PMC13593758; doi:10.3389/fpubh.2026.1850011)
Supplement: Supplementary file 5 [file Table_4.DOCX]

**Supplementary Table S1.** Comparison of Clinical Data Between the Training and Internal Validation Cohorts

| Variable | Training Set  (n=214) | Internal Validation Set (n=90) | *p*-value |
| --- | --- | --- | --- |
| Sex |  |  | 0.887 |
| Male, n(%) | 165 (77%) | 68 (76%) |  |
| Education Level |  |  | 1.000 |
| ≤ Primary school,n (%) | 175 (82%) | 74 (82%) |  |
| Use of Quinolones |  |  | 1.000 |
| Yes, n(%) | 32 (15%) | 13 (14%) |  |
| Age, years | 76.00 (71.00, 81.75) | 75.50 (71.00, 81.00) | 0.539 |
| BMI (kg/m²) | 21.26 ± 3.60 | 20.56 ± 4.16 | 0.170 |
| Smoking Duration,years | 30 (0, 50) | 40 (0, 40) | 0.427 |
| Cigarettes per Day | 20 (0, 40) | 20 (0, 40) | 0.854 |
| mMRC Grade |  |  | 0.491 |
| Grade 0, n(%) | 0 (0%) | 0 (0%) |  |
| Grade 1, n(%) | 39 (18%) | 20 (22%) |  |
| Grade 2, n(%) | 45 (21%) | 21 (23%) |  |
| Grade 3, n(%) | 58 (27%) | 17 (19%) |  |
| Grade 4, n(%) | 72 (34%) | 32 (36%) |  |
| WBC (×10⁹/L) | 7.48 (5.56, 10.18) | 7.97 (6.46, 10.45) | 0.132 |
| Neu (%) | 79.55 (70.82,84.88) | 81.25 (74.17, 86.47) | 0.068 |
| NEU (×10⁹/L) | 5.87 (4.17, 8.37) | 6.14 (4.71, 9.33) | 0.139 |
| RBC (×10¹²/L) | 4.13 ± 0.66 | 4.20 ± 0.63 | 0.349 |
| Hb (g/L) | 126 (113, 136) | 127 (116.25, 137) | 0.383 |
| PLT (×10⁹/L) | 176(139.25,229.50) | 186.50(143.50,236.75) | 0.317 |
| PT (s) | 11.30 (10.80,12.00) | 11.25 (10.60,12.10) | 0.407 |
| PT% (%) | 89.30 (80.00,95.38) | 89.50 (81.30, 97.75) | 0.425 |
| APTT (s) | 27.85 (26.13,29.60) | 28.40 (25.83, 30.50) | 0.246 |
| FIB (g/L) | 4.11 (3.27, 5.49) | 4.11 (3.31, 5.26) | 0.780 |
| TT (s) | 17.60 (16.80,18.58) | 17.60 (16.83, 18.60) | 0.420 |
| D-Dimer (μg/ml) | 0.58 (0.33, 1.06) | 0.66 (0.42, 1.16) | 0.052 |
| ALT (U/L) | 17 (12, 26.75) | 16 (13, 22) | 0.446 |
| AST (U/L) | 22 (18, 29.75) | 23 (18, 31) | 0.536 |
| TBIL (μmol/L) | 12.90 (9.00, 17.85) | 12.40 (9.25, 16.15) | 0.699 |
| TP (g/L) | 65.00 (60.40,70.07) | 65.30 (60.08, 71.00) | 0.694 |
| ALB (g/L) | 38.71 ± 4.44 | 38.81 ± 4.07 | 0.844 |
| Urea (mmol/L) | 6.85 (5.20, 9.20) | 7.25 (5.75, 9.28) | 0.442 |
| Creatinine(μmol/L) | 65.30 (55.00,84.05) | 63.00 (52.33, 82.92) | 0.327 |
| eGFR(ml/min/1.73m²) | 93.30 (81.07,99.85) | 93.30 (85.18, 101.52) | 0.499 |
| Uric Acid (μmol/L) | 304.50(236.25,394.00) | 278.00 (215.50, 348.75) | 0.060 |
| GLU (mmol/L) | 5.80 (5.00, 6.88) | 6.00 (5.10, 7.97) | 0.233 |
| K⁺ (mmol/L) | 4.05 ± 0.57 | 3.99 ± 0.61 | 0.412 |
| Na⁺ (mmol/L) | 140.60(137.65,142.28) | 140.35(137.95,142.40) | 0.826 |
| Stigma (score) | 52 (49, 55) | 52 (49, 54) | 0.439 |
| PHYS (score) | 11.43 (9.71, 13.00) | 11.43 (10.29, 13.14) | 0.726 |
| PSYCH (score) | 12.00 (11.33, 13.33) | 12.00 (11.33, 12.67) | 0.990 |
| SOCIL (score) | 12.00 (12.00, 12.00) | 12.00 (12.00, 12.00) | 0.906 |
| ENVIR (score) | 13.00 (11.50, 13.50) | 13.00 (11.50, 14.00) | 0.990 |
| Quality of Life, n (%) |  |  | 0.442 |
| 1 | 1 (0%) | 0 (0%) |  |
| 2 | 34 (16%) | 10 (11%) |  |
| 3 | 120 (56%) | 54 (60%) |  |
| 4 | 59 (28%) | 26 (29%) |  |
| 5 | 0 (0%) | 0 (0%) |  |
| Health Status, n (%) |  |  | 0.205 |
| 1 | 2 (1%) | 1 (1%) |  |
| 2 | 106 (50%) | 39 (43%) |  |
| 3 | 94 (44%) | 40 (44%) |  |
| 4 | 12 (6%) | 10 (11%) |  |
| 5 | 0 (0%) | 0 (0%) |  |
| Comorbidities |  |  | 0.195 |
| Yes, n(%) | 144 (67%) | 68 (76%) |  |
| Annual Hospitalizations, times | 1 (1, 2) | 1 (1, 2) | 0.960 |
| Hospital Stay, days | 9 (7, 10) | 8 (7, 9) | 0.092 |
| Use of Theophylline |  |  | 0.287 |
| Yes, n(%) | 177 (83%) | 69 (77%) |  |
| Use of Corticosteroids |  |  | 0.008 |
| No, n(%) | 22 (10%) | 1 (1%) |  |
| Inhaled only, n(%) | 37 (17%) | 11 (12%) |  |
| Systemic, n(%) | 155 (72%) | 78 (87%) |  |

**Note:** Comorbidities refer to hypertension, diabetes mellitus, coronary atherosclerotic heart disease, and cerebral infarction. Abbreviations: WBC=White Blood Cell Count; Neu=Neutrophil Percentage; NEU=Absolute Neutrophil Count; RBC=Red Blood Cell Count; Hb=Hemoglobin; PLT=Platelet Count; PT=Prothrombin Time; PT%=Prothrombin Time Activity; APTT=Activated Partial Thromboplastin Time; FIB=Fibrinogen; TT=Thrombin Time; D-Dimer=D-Dimer; ALT=Alanine Aminotransferase; AST=Aspartate Aminotransferase; TBIL=Total Bilirubin; TP=Total Protein; ALB=Albumin; Urea=Urea; Creatinine=Creatinine; eGFR=Estimated Glomerular Filtration Rate; Uric Acid=Uric Acid; GLU=Blood Glucose; K+=Potassium; Na+=Sodium. *p* <0.05 was considered statistically significant.
