# Supplementary material for: Development and temporal prospective validation of an interpretable machine learning model for identifying depressive symptoms in patients with acute exacerbation of chronic obstructive pulmonary disease
Source: Front Public Health. 2026 Sep 8;14:1850011. doi: 10.3389/fpubh.2026.1850011 (PMC13593758; doi:10.3389/fpubh.2026.1850011)
Supplement: Supplementary file 6 [file Table_5.DOCX]

**Supplementary Table S2.** Core performance metrics of various machine learning models on the training set.

| ML | Accuracy | Recall | Precision | Specificity | F1 score | AUC | Brier score |
| --- | --- | --- | --- | --- | --- | --- | --- |
| DT | 0.762 | 0.765 | 0.677 | 0.760 | 0.718 | 0.816 | 0.152 |
| PLS | 0.565 | 0.988 | 0.477 | 0.287 | 0.644 | 0.866 | 0.178 |
| GBM | 0.808 | 0.941 | 0.690 | 0.721 | 0.796 | 0.910 | 0.119 |
| SVM | 0.743 | 0.882 | 0.625 | 0.651 | 0.732 | 0.866 | 0.146 |
| LR | 0.762 | 0.871 | 0.649 | 0.690 | 0.744 | 0.866 | 0.146 |
| NB | 0.752 | 0.847 | 0.643 | 0.690 | 0.731 | 0.863 | 0.159 |
| RF | 0.911 | 0.976 | 0.830 | 0.868 | 0.897 | 0.995 | 0.049 |
| SVC | 0.780 | 0.847 | 0.679 | 0.736 | 0.754 | 0.861 | 0.151 |
| XGB | 0.790 | 0.929 | 0.669 | 0.698 | 0.778 | 0.916 | 0.116 |
| KNN | 0.762 | 0.929 | 0.637 | 0.651 | 0.756 | 0.887 | 0.135 |
