# Supplementary material for: Development and temporal prospective validation of an interpretable machine learning model for identifying depressive symptoms in patients with acute exacerbation of chronic obstructive pulmonary disease
Source: Front Public Health. 2026 Sep 8;14:1850011. doi: 10.3389/fpubh.2026.1850011 (PMC13593758; doi:10.3389/fpubh.2026.1850011)
Supplement: Supplementary file 7 [file Table_6.DOCX]

**Supplementary Table S3.** Core performance metrics of different stacking ensemble models on the training set.

| ML | Accuracy | Recall | Precision | Specificity | F1 score | AUC | Brier score |
| --- | --- | --- | --- | --- | --- | --- | --- |
| LR+SVM | 0.757 | 0.824 | 0.654 | 0.713 | 0.729 | 0.863 | 0.149 |
| NB+SVM | 0.748 | 0.824 | 0.642 | 0.698 | 0.722 | 0.848 | 0.157 |
| LR+NB | 0.776 | 0.835 | 0.676 | 0.736 | 0.747 | 0.864 | 0.151 |
| LR+NB+SVM | 0.748 | 0.847 | 0.637 | 0.682 | 0.727 | 0.854 | 0.154 |

**Note:** Column names indicate the stacking model combinations: LR+SVM, LR+NB, NB+SVM, and LR+NB+SVM.
