# Supplementary material for: Development and temporal prospective validation of an interpretable machine learning model for identifying depressive symptoms in patients with acute exacerbation of chronic obstructive pulmonary disease
Source: Front Public Health. 2026 Sep 8;14:1850011. doi: 10.3389/fpubh.2026.1850011 (PMC13593758; doi:10.3389/fpubh.2026.1850011)
Supplement: Supplementary file 8 [file Table_7.DOCX]

**Supplementary Table S7.** Comparison of predictive performance between logistic regression and SVM+LR stacking models.

| Cohort | Model | AUC | Brier | Accuracy | Recall | Specificity |
| --- | --- | --- | --- | --- | --- | --- |
| Training | LR | 0.866(0.819-0.912) | 0.146 | 0.762 | 0.871 | 0.690 |
| Training | SVM | 0.866(0.813-0.910) | 0.146 | 0.743 | 0.882 | 0.651 |
| Training | PLS | 0.866(0.814-0.912) | 0.178 | 0.565 | 0.988 | 0.287 |
| Training | SVM+LR | 0.863(0.812-0.909) | 0.149 | 0.757 | 0.824 | 0.713 |
| Internal validation | LR | 0.862(0.787-0.930) | 0.151 | 0.767 | 0.861 | 0.704 |
| Internal validation | SVM | 0.863(0.786-0.927) | 0.152 | 0.744 | 0.861 | 0.667 |
| Internal validation | PLS | 0.867(0.789-0.936) | 0.183 | 0.544 | 1.000 | 0.241 |
| Internal validation | SVM+LR | 0.868(0.792-0.936) | 0.154 | 0.778 | 0.806 | 0.759 |
| Prospective validation | LR | 0.866(0.772-0.960) | 0.098 | 0.883 | 0.800 | 0.911 |
| Prospective validation | SVM+LR | 0.864(764-961) | 0.098 | 0.892 | 0.733 | 0.944 |
