# Supplementary material for: Development and temporal prospective validation of an interpretable machine learning model for identifying depressive symptoms in patients with acute exacerbation of chronic obstructive pulmonary disease
Source: Front Public Health. 2026 Sep 8;14:1850011. doi: 10.3389/fpubh.2026.1850011 (PMC13593758; doi:10.3389/fpubh.2026.1850011)
Supplement: Supplementary file 9 [file Table_8.DOCX]

**Supplementary Table S8.** Threshold sensitivity analysis of the final logistic regression model in the prospective validation cohort (n = 120).

| Threshold | Sensitivity (%) | Specificity (%) | PPV (%) | NPV (%) | Accuracy (%) | Net benefit |
| --- | --- | --- | --- | --- | --- | --- |
| 0.10 | 80.0 | 67.8 | 45.3 | 91.0 | 70.8 | 0.1731 |
| 0.15 | 80.0 | 74.4 | 51.1 | 91.8 | 75.8 | 0.1662 |
| 0.20 | 80.0 | 74.4 | 51.1 | 91.8 | 75.8 | 0.1521 |
| 0.25 | 80.0 | 85.6 | 64.9 | 92.8 | 84.2 | 0.1639 |
| 0.30 | 80.0 | 91.1 | 75.0 | 93.2 | 88.3 | 0.1714 |
| 0.35 | 70.0 | 95.6 | 84.0 | 90.5 | 89.2 | 0.1571 |
| 0.40 | 66.7 | 96.7 | 87.0 | 89.7 | 89.2 | 0.1500 |
| 0.45 | 60.0 | 97.8 | 90.0 | 88.0 | 88.3 | 0.1364 |
| 0.50 | 60.0 | 97.8 | 90.0 | 88.0 | 88.3 | 0.1333 |

**Note:**PPV = positive predictive value; NPV = negative predictive value. Net benefit was calculated using decision curve analysis. The Youden index–derived cutoff in this cohort was 0.2837.
